# Whole transcriptome sequencing from *Zanthoxylum armatum*: implications on metabolic pathway analysis and regulation

**DOI:** 10.3389/fpls.2026.1795497

**Published:** 2026-06-11

**Authors:** Moirangthem Lakshmipriyari Devi, Khomdram Khedashwori Devi, Khundrakpam Basanti, Sudripta Das

**Affiliations:** Plant Molecular Genetics and Genomics, Plant Bioresources, Institute of Bioresources and Sustainable Development (IBSD), Imphal, Manipur, India

**Keywords:** correlation matrix, *de novo* transcriptome, isoquinoline alkaloids, qRT-PCR, *Zanthoxylum armatum*

## Abstract

*Zanthoxylum armatum*, a deciduous aromatic shrub has been utilized by traditional healers for treatment of various ailments. Elucidation of the transcriptome data and expression studies of the putative biosynthetic pathway gene(s) of berberine and sanguinarine production in leaf, stem, and fruit at different seasons and identification of transcription factor families involved in the biosynthesis of isoquinoline alkaloids was established in the present study. The assembled transcripts were clustered into 44254, 46402 and 46521 unigenes and a total of 32118, 27777 and 19754 CDS were predicted from unigenes in fruit, leaf and stem samples respectively. Using MISA 5576 SSRs were identified from fruit, leaf and stem samples, out of which a total of 1877 SSRs with 150 flanking regions were predicted. Putative biosynthetic pathway genes like *BBE*, *BBE-like*, *SOMT*, *CAS*, *STOX*, *BS* and *SR* were significantly expressed in the three samples of the plant in different seasons and at different level. Transcription factor analysis along with correlation matrix predicted abundant families like *AP2/ERF* family (4010), followed by *MYB-*related family (3010), *RPL2 family* (2760), *MYC family* (2662), *DREB/CRF family* (2526), and *RAV* (2302) to be the regulators along with WRKY. The association analysis of the metabolome and the assembled transcriptome from *Zanthoxylum armatum* will go a long way in re-engineering of the species by AI assisted priming. The TFs would enable artificial intelligence-based designing of efficient minipromoters for enhancing the production of the target compounds in a more efficient and predictable manner.

## Introduction

The genus *Zanthoxylum* L. is a deciduous shrub or tree that belong to the Rutaceae family and has 250 species native to the warm, subtropical and temperate regions of the world (Negi et al., 2011). This genus includes trees and shrubs which are usually dioecious (Patiño et al., 2012). According to the World Online (POWO) database, there are 234 species of *Zanthoxylum* L (POWO, 2024). In India, approximately 12 species of this genus have been documented: *Z*. *acanthopodium*, *Z*. *armatum*, *Z*. *burkillianum*, *Z*. *myriacanthum, Z*. *nitidum*, *Z*. *ovalifolium*, *Z*. *oxyphyllum*, *Z*. *pseudoxyphyllum*, *Z*. *rhetsa*, *Z*. *scandens*, *Z*. *tetraspermum and Z*. *tomentellum* (Hajra et al., 1997).

This genus is economically important owing to its medicinal, alimentary and industrial applications. Different species of *Zanthoxylum* have been used in folk medicine for myocardium disorder attenuation, bone injury alleviation, and cold resistance (Chung et al., 2006), as analgesic and anti-inflammatory drugs in Chinese folk medicine (Guo et al., 2011) and used as folk medicine to relieve toothache and sore throat (Yang et al., 2008). *Zanthoxylum armatum* var. *novemfolius* male and female floral transcriptomes revealed that transcription factors such as *AGL11*, *PMADS2*, and *NAC* played crucial roles in flower development (Zhang et al., 2020). Transcriptome assembly of the plant organs (roots, stems, leaf at budding stage and mature, and fruits) identified genes related to metabolism of fatty acids and terpenoids (Hui et al., 2020). A subsequent study screened genes that were significantly differentially expressed in the fruit compared to other organs (root, stem, leaves, and bud), enriched in 17 KEGG pathways, and identified the important genes linked to the biosynthesis of terpenoids (28 genes), alkaloids (5 genes), and flavonoids (7 genes) in *Z*. *armatum* fruit (Wenkai et al., 2021). Berberine and sanguinarine are benzylisoquinoline alkaloids (BIA’s) belonging to the protoberberine-type and benzophenanthridine, respectively, and their biosynthetic pathways have been studied in *Argemone mexicana* L. (Mexican poppy) (Vázquez-Flota et al., 2018). *Papaver somniferum* (opium poppy) has been studied for the molecular characterization of berberine bridge enzyme genes (Facchini et al., 1996b) and the enzyme was cloned, expressed and induced in a study in *Eschscholzia californica* (California poppy) which showed importance during the formation of benzophenanthridine alkaloids due to pathogenic attack (Dittrich and Kutchan, 1991).

Berberine is an important active ingredient of *Coptis chinensis* and has traditionally been used in clinical treatment. A novel (*S*)-canadine synthase gene (*CcCAS-1*) was isolated from the plant which is essential for berberine biosynthesis in *C*. *chinensis* (He et al., 2015). Cloning and characterization of *S*-Adenosyl-L-methionine: scoulerine-9-*O*-methyltransferase (SMT), which catalyzes the transfer of the S-methyl group during the biosynthesis of berberine has been performed in cultured cells of *Coptis japonica* (Takeshita et al., 1995).

Sanguinarine is an antimicrobial alkaloid commonly found in plants of the Papaveraceae family including *Papaver somniferum* roots. A cDNA encoding a key enzyme in sanguinarine biosynthesis (*S*)-*cis*-N-methylstylopine 14-hydroxylase, was isolated and characterized from the opium poppy (Beaudoin and Facchini, 2013). The biosynthesis of benzo[c]phenanthridine alkaloids was investigated in a cell cultures of *Macleaya cordata* (Papaveraceae) (Takao et al., 1983). In the present study, leaves, stems and fruits of plants at the green young fruit stage collected in summer were sequenced for *de novo* transcriptome analysis to identify pathway genes related to biosynthesis and related transcription factor families.

Numerous studies have employed RNA-seq to investigate transcriptional profiles and provide insights into various traits. These include chromosome-scale genome analysis (Miao et al., 2024), pigmentation (Wang et al., 2020) and drought tolerance (Dossa et al., 2017). Zhang et al. (2024) conducted transcriptomic analysis of low-nitrogen tolerance. Wang et al. (2019) examined transcriptomes with contrasting oil contents, and Zhang et al. (2021) analyzed the miRNAs involved in fatty acid biosynthesis. Pamei and Makandar (2022) performed a comparative RNA-seq analysis to study floral regulation and defense responses against phytoplasmas.

Studies on mutant *Arabidopsis* lines regulating fatty acid desaturation or phenylpropanoid metabolism have revealed exclusive changes in lignan and fatty acid profiles. Specific transcription factors such as MYB and bZIP families, control the activity of genes linked to both lignan production and lipid metabolism in *Arabidopsis thaliana*, suggesting a unified regulatory system governing these processes (Kelly, 2018). These metabolic pathways usually intersect by sharing key precursors and cofactors, resulting in significant metabolic interplay. In *Arabidopsis thaliana*, peroxidases and cytochrome P450s play important roles in both fatty acid metabolism and modification processes within the phenylpropanoid pathway (Mentzen et al., 2008).

In the present study, validation of the transcriptome data and expression studies of the putative pathway gene(s) of berberine and sanguinarine biosynthesis in leaves, stems, and fruits and their expression variation due to seasonal changes and identification of transcription factors (TFs) and their correlation coefficients were established.

## Materials and methods

### Sample collection

**Figure 1:**
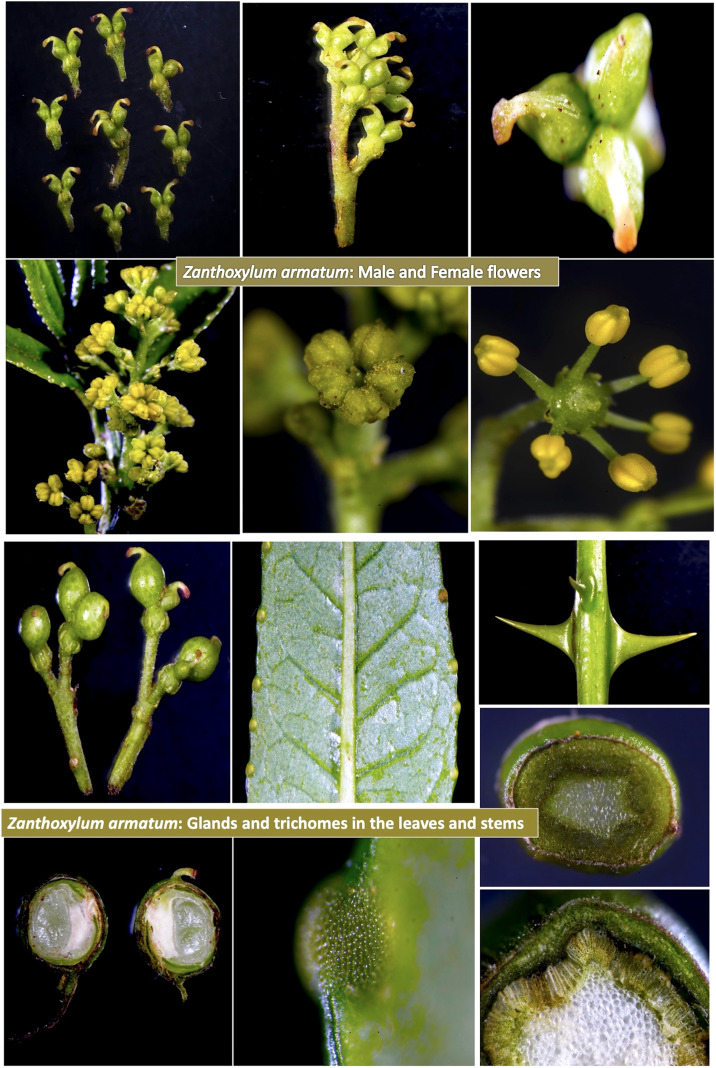
*Zanthoxylum armatum* DC. male plants and female plants with organs, with distinct oil dots on the margin of the leaflets, stem with young leaves.

Leaves, stems and fruits of *Z*. *armatum* were collected during spring, green in summer, and mature fruit stage in monsoon and winter (**Figure 1**). Samples were collected from the leaves, stems and fruits of the plants to prepare pooled samples. The samples were dissolved in RNAlater^®^ Solution (Invitrogen) and kept overnight at 4 °C and transferred to -80 °C (**Supplementary Figures 1**, **2**).

The leaf, stem and fruit samples were used for transcriptome sequencing. Samples collected during the spring, summer, monsoon and winter were used in the expression analysis of putative biosynthetic pathway gene(s) of alkaloids such as berberine and sanguinarine. There were 3 biological replicates with 3 technical replicates per biological replicate.

Total RNA from the pooled samples was isolated using RNeasy Plant Mini Kit (Qiagen, Germany). The isolated RNA was run on 1% formaldehyde denaturing agarose gel using 1X MOPS buffer and quantified using Nanodrop (Shimadzu, Japan) and Qubit^®^ 2.0 fluorometer (Invitrogen).

### Sequencing of Illumina 2 x 150 PE library

The libraries were prepared from QC passed samples with input total RNA ~1μg using the Illumina TruSeq Stranded mRNA Library Preparation Kit (Illumina) according to the manufacturer’s protocol. Briefly, total RNA was enriched with Oligo dT beads for mRNA fragments and then subjected to purification, fragmentation, and priming for cDNA synthesis. The fragmented mRNA was converted into first-strand cDNA, followed by second-strand cDNA synthesis, A-tailing, adapter ligation, and amplification by the recommended number of PCR cycles. The quality and quantity of library were checked using an Agilent DNA High Sensitivity Assay Kit (**Supplementary Figure 3**).

After obtaining the required concentration and the mean peak size from the Bioanalyzer profile, the library was loaded into the Illumina platform for cluster generation and sequencing. Paired-end sequencing allows the template fragments to be sequenced in both the forward and reverse directions. The library molecules bind to complementary adapter oligos in paired-end flow cells. The adapters were designed to allow selective cleavage of the forward strands after the re-synthesis of the reverse strand during sequencing. Adapter sequences and poor quality reads (<Q30) were removed using BBduk (Bushnell et al., 2017). Next, cleaned data were fed into STAR (Dobin et al., 2013) for alignment. FeatureCounts (Liao et al., 2014) was used for the quantification of aligned reads. The copied reverse strand was then used to sequence from the opposite end of the fragment. The sequencing was performed on the Illumina HiSeq 2500 platform at Xcelris Labs Limited (Ahmedabad, India). The raw sequences were submitted to the National Center for Biotechnology Information (NCBI) as well as to IBDC, India (**Supplementary Figure 3**).

### *De novo* assembly and unigenes and CDS prediction from master assembly

Master assembly was performed by taking reads of fruit, leaf and stem samples together using Trinity v2.8.5 (at default parameters, kmer 25). Transcripts were further distributed performed according to their length. Statistical elements of the assembly were calculated using Perl scripts. Transcripts were further processed for unigenes prediction using CD-HIT package v4.8.1. CD-HIT-EST executable was used to remove the shorter redundant transcripts when they were completely covered by other transcripts with greater than 90% identity (default parameter). The non-redundant clustered transcripts were then termed as unigenes. Further distribution of unigenes was performed according to length (**Supplementary Figures 4-5**).

CDS were predicted from the unigene sequences using TransDecoder v5.5.0 using default parameters with encoded protein length set a minimum of 100 amino acids, and homology search with Swiss-Prot and Pfam databases. Further distribution of CDS was performed according to length (**Supplementary Figure 6**).

The protein sequences corresponding to the predicted coding regions within the unigenes were subjected to a similarity search against NCBI’s non-redundant (NR) database using the BLASTP algorithm with an e-value threshold of 1e-5. Simultaneously, protein sequences were searched for similarity against the Uniprot, KOG, and Pfam databases using BLASTP with an e-value threshold of 1e-5. BLAST v2.8.1 was used.

### GO sequence distribution of NR annotated CDS/proteins and pathway analysis

The Gene Ontology project provides controlled vocabularies of defined terms that represent gene-product properties. GO mapping was performed to retrieve GO terms for all the BLAST annotated proteins against the NR database using Blast2GO CLI 1.4.1. and WEGO (Web Gene Ontology Annotation Plot), which is a useful tool for plotting GO annotation results were used to represent GO categories. To comprehensively obtain the functional information, all genes were aligned by BLASTX against NCBI nonredundant protein sequence (NR), NCBI nucleotide sequence (NT), Protein family (Pfam), KEGG Orthology (KO) and euKaryotic Ortholog Groups (KOG) databases with a threshold E-value of 10−5. Meanwhile, the functional annotation and classification of all genes were also conduct based on Gene Ontology database2 (GO) and Kyoto Encyclopedia of Genes and Genomes database3 (KEGG). Ortholog assignment and mapping of CDS to the biological pathways were performed using the KEGG automatic annotation server (KAAS). All the CDS were compared against KEGG database using BLASTP with a threshold bit-score value of 60 (default).

### SSR identification from CDS

Simple sequence repeats (SSRs) or microsatellites are tandem repeats of nucleotide motifs of sizes 2–6 bp and are highly polymorphic and ubiquitously present in all the known genomes. Thus, SSR were identified in the CDS sequences of each sample using with the MISA perl script. The criteria used for SSR identification were as follows: Adinucleotide pattern should appear at least six times, trinucleotide pattern >=five times, tetra nucleotide pattern >=five times, pentanucleotide pattern >=five times, and hexanucleotide pattern >=five times. SSRs with a flanking of 150 bp (upstream and downstream) were fetched with Python script and primers were designed.

### *In silico* expression analysis

The combinations considered for the DEG analysis were Leaf vs Stem, Leaf vs Fruit and Stem vs Fruit. The reads of each sample were mapped separately to the CDS sequences obtained from the master assembly using BWA v0.7.12-r1039. Duplicate PCR reads were removed from the alignment file using Picard’s MarkDuplicates.jar software. The mapped reads in the mentioned combination were considered for DGE analysis. The read mapped count from each sample was provided as an input for differential expression analysis using the edgeR bioconductor package in R.

Differential gene expression was inferred between sample groups using the R package edgeR v3.28.1. edgeR is a bioconductor package based on the negative binomial distribution method.

Highly significant 50 CDS based on q-values with proper annotations, were selected for heatmap generation. The pheatmap package from the R software was used to generate a heatmap. The color coding ranges from red to blue, where red represents high transcript expression and blue represents low transcript expression. The MA plot visualizes the differences between measurements taken in two samples by transforming the data onto M (log ratio) and A (mean average) scales and then plotting the obtained values. The “volcano plot” arranges expressed genes along dimensions of biological as well as statistical significance.

### Identification of transcription factor

To identify transcription factors, protein sequences were searched for similarity against the PlantTFDB v5 database using BLASTP with an e-value cut-off of 1e-05.

### qRT-PCR of expressed sequences and validation

Semi-quantitative PCR was performed to screen and check the efficiency of the primers for transcriptome data validation and to identify the putative biosynthetic pathway gene(s)of berberine and sanguinarine in the samples collected in different seasons. All the up- or down- regulations in the following description represent in the first comparison component. The differential expression genes (DEGs) among each comparison were detected by DESeq2 package (1.16.1). Foldchange (FC) was the gene expression difference between different samples, and the threshold was set as |log2 (FC)| > 1 and p-value < 0.05 to identify the DEGs. Primers were designed using Primer3Plus (https://www.bioinformatics.nl/cgi-bin/primer3plus/primer3plus.cgi) except for GAPDH and 18S rRNA which were used as reference genes. Total RNA was extracted from the samples using the RNeasy^®^ Plant Mini Kit (Qiagen, Germany) and concentrated using an RNA Clean & Concentrator™-25 (Zymo Research, United States). cDNA was synthesized using a High-Capacity cDNAReverseTranscription Kit with RNase Inhibitor (Applied Biosystems, ThermoFisher Scientific). Reaction mixture (Promega, USA) of 10 µL comprised of PCR master mix (2X)- 5 µL, forward primer (10 µM)- 0.5 µL, reverse primer (10 µM)- 0.5 µL, cDNA (10 ng/µL)- 1 µL, nuclease free water- 3 µL. The PCR conditions used were, step 1-initial denaturation at 94 °C (60 secs); step 2- denaturation at 94 °C (60 secs); step 3 annealing at 60 °C (40 secs); step 4 extension at 72 °C (40 secs); step 2-4–35 cycles; step 5 final extension 72 °C (5 min). PCR was performed using a Bio-Rad C1000 Touch Thermal Cycler. Hierarchical clustering was performed using the SciPy function “scipy.cluster.hierarchy.linkage” using “average” method, with a distance metric of 1 − abs (*ρij*), where *ρij* is the Pearson correlation between genes i and j.

### Preparation for HPLC and analysis

The standards were weighed carefully and dissolved in methanol to prepare a standard solution of 1 mg/mL and pass-through syringe filter of 0.45 and 0.22 µm (BR Biochem, India) and further diluted to prepare required concentrations for calibration curve for HPLC analysis. Standard solutions (1, 2, 6, 10, and 14 µg/mL) were prepared for the calibration curve. The solutions were stored at 4 °C in a refrigerator until use (Devi, 2025). For quantitatively analyze the alkaloids, 10 mg/mL of the ethanolic extracts of the samples were prepared using HPLC-grade methanol (Merck, India) and filtered through 0.45 and 0.22 µm syringe filters (BR Biochem, India). Spike solutions were also prepared for selected samples with different concentrations of berberine (8, 10, 12, 16 and 18 µg/mL) and sanguinarine (4, 6, 8, 12 and 18 µg/mL) for validation purpose (Devi, 2025). The instrument used for chromatography was a Shimadzu Prominence High Performance Liquid Chromatograph system (Japan, Model CBM-20A, LC-20AD, SPD-M20A) with a diode array detector (photodiode array). The analytical column used for separation was Shim-pack GIST C18 (particle size 5µm, 4.6 x 250 mm, Shimadzu, Japan). Acetonitrile and 0.1% trifluoroacetic acid (TFA) (60:40 v/v) were used as mobile phases which was then degassed and sonicated. The flow rate was set at 1mL/min and the injection volume was 10µL at a set temperature of 35 °C. The wavelength ranges from 190 to 800 nm, and the slit width was 1.2 nm. The analyses were performed under isocratic conditions. The chromatographic data were recorded and processed using LC Solutions software v1.25 (Devi, 2025). HPLC data were analyzed using two-way ANOVA followed by Tukey’s multiple comparison *post hoc* test using GraphPad Prism v8.4.3 (686). Values were considered statistically significant at *p* < 0.05.

## Results

### RNA extraction and library preparation

A paired-end library was prepared from total RNA using the Illumina TruSeq Stranded mRNA Library Preparation Kit (**Supplementary Figure 1**). Amplified libraries were analyzed on a Bioanalyzer 2100 (Agilent Technologies) using a high-sensitivity (HS) DNA chip according to the manufacturer’s instructions. The mean size of the libraries was 401bp, 419bp and 413bp for leaf, stem and fruit respectively (**Supplementary Figure 2**). The data statistics of the next-generation sequencing and the software used are provided in **Tables 1** and **2**. The NCBI BioProject ID of the samples was PRJNA1146397, and the BioSample accessions were SAMN43101982 (leaf), SAMN43101983 (stem), and SAMN43101984 (fruit).

**Table 1:** Statistical data for transcriptome sequencing of tree types of samples viz. fruit, leaf and stem of *Zanthoxylum armatum* DC.

Sample ID	# PE reads	Total reads (P1+P2)	Total bases (R1+R2)	Total data (Gb)
Fruit	22741590	45483180	6712655898	6.7
Leaf	23663721	47327442	6995718090	7.0
Stem	19990282	39980564	5931482327	5.9

**Table 2:** List of software utilized for analysis of the transcriptome of *Zanthoxylum armatum*.

Software	Version	Application
*Trinity*	2.8.5	RNASeq *denovo* assembly
*Cd-hit*	4.8.1	Transcript clustering to generate unigenes
*TransDecoder*	5.5.0	CDS prediction from unigenes
*Blast*	2.8.1	Functional annotation of proteins against NR, Uniprot, KOG, Pfam and Transcription factor database
*Blast2GO cli*	1.4.1	Gene Ontology (GO) mapping and annotation
*KAAS*	Webserver	Pathway analysis against KEGG database
*MISA*	A perl script	SSR identification
*BWA*	0.7.12-r1039	Mapping of reads to CDS for Expression profiling
*Picard-tools*	1.60	Removing multi-mapped reads from BAM file
*Samtools*	1.4.1	Reads mapped count
*R package*	3.6.2	Differential expression analysis and its visualization

### *De novo* assembly, unigenes and CDS prediction from master assembly

The statistical elements of the assembly were calculated using in-house Perl scripts, listed in **Table 1**. Transcripts were processed for unigene prediction using the CD-HIT package. CD-HIT-EST executable and the shorter redundant transcripts were removed when they were completely covered by other transcripts with more than 90% identity (default parameter). The non-redundant clustered transcripts were then named unigenes. Fasta sequences of predicted unigenes are provided in the data deliverables folder entitled “02_Unigene_sequences”. The basic statistics for the predicted unigenes are listed in **Table 3**. Annotation analysis identified 17 CDS from 17 unigenes for the putative biosynthetic pathway gene, berberine bridge enzyme, and two CDS from two unigenes for the putative biosynthetic pathway gene, sanguinarine reductase. Further distribution of the unigenes was performed according to their length (**Tables 4**–**6**; **Supplementary Figures 4**–**6**). The protein sequences from the predicted coding regions within the unigenes were searched against the NCBI’s non-redundant (nr) database using the BLASTP algorithm with an e-value threshold of 1e-5 (**Table 6**). The top-hit species distribution revealed that the majority of the hits were against the species *Citrus sinensis* followed by *Citrus clementina* (**Supplementary Figure 7**). Simultaneously all protein sequences were searched for similarity against the Uniprot, KOG and Pfam database using BLASTP with an e-value threshold of 1e-5. Results of the similarity search against all four databases are given in **Table 7**.

**Table 3:** Statistical data of Master assembly of transcripts obtained from *de novo* RNA-seq of *Zanthoxylum armatum*.

Description	Master assembly
*Total number of transcripts*	164899
*Total transcriptome size (bp)*	195937123
*Average transcript length (bp)*	1188
*Maximum transcript length (bp)*	18443
*Minimum transcript length (bp)*	300
*Transcript N50 (bp)*	1848

**Table 4:** Statistical data of unigenes identified from master assembly of transcripts of *Z. armatum*.

Description	Master unigene
*Total number of unigenes*	137177
*Total number of bases in unigenes (bp)*	152048042
*Mean unigene length (bp)*	1108
*Maximum unigene length (bp)*	18443
*Minimum unigene length (bp)*	300
*Unigene N50 (bp)*	1727

**Table 5:** Statistical data of CDS identified from master assembly of transcripts of *Z. armatum*.

Description	Master CDS
*Total number of CDS*	79649
*Total number of bases in CDS (bp)*	70755312
*Mean CDS length (bp)*	888
*Maximum CDS length (bp)*	10425
*Minimum CDS length (bp)*	255

**Table 6:** Statistics data of Blast of transcripts against NR, Uniprot, KOG and Pfam databases.

Blast database	Master CDS
*Total*	79649
*NR*	74049
*UniProt*	60331
*KOG*	38489
*Pfam*	38111

**Table 7:** KOG Classification for CDS obtained from RNA-seq of *Z. armatum*.

Description	Class	Master CDS
*RNA processing and modification*	A	1996
*Chromatin structure and dynamics*	B	664
*Energy production and conversion*	C	1645
*Cell cycle control, cell division, chromosome partitioning*	D	939
*Amino acid transport and metabolism*	E	1601
*Nucleotide transport and metabolism*	F	493
*Carbohydrate transport and metabolism*	G	2195
*Coenzyme transport and metabolism*	H	542
*Lipid transport and metabolism*	I	1741
*Translation, ribosomal structure and biogenesis*	J	2137
*Transcription*	K	2704
*Replication, recombination and repair*	L	1170
*Cell wall/membrane/envelope biogenesis*	M	617
*Cell motility*	N	15
*Post-translational modification, protein turnover, chaperones*	O	4340
*Inorganic ion transport and metabolism*	P	1323
*Secondary metabolites biosynthesis, transport and catabolism*	Q	1549
*General function prediction only*	R	5442
*Function unknown*	S	2458
*Signal transduction mechanisms*	T	5668
*Intracellular trafficking, secretion and vesicular transport*	U	2287
*Defense mechanisms*	V	347
*Extracellular structures*	W	151
*Nuclear structure*	Y	125
*Cytoskeleton*	Z	1098

KOG analysis showed that the most enriched KOG category was “Signal transduction mechanisms (T),” “General function prediction only (R),” and “Posttranslational modification, chaperones (O)” (**Supplementary Figures 7**, **9**). In Pfam analysis, most abundant domains identified were “Protein kinase domain” followed by “Protein tyrosine kinase.” A comparative account of CDS annotations and comparative numbers in different databases was represented in the form of a Venn diagram using the InteractiVenn online server (http://www.interactivenn.net/) (**Supplementary Figure 10**).

GO mapping was performed to retrieve GO terms for all BLASTP functionally annotated proteins against the NR database using Blast2GO 1.4.1. CDS were assigned to least one GO term (a single CDS can have more than one GO term) (**Tables 8**, **9**; **Supplementary Figure 11**). Web Gene Ontology Annotation Plot (WEGO), which is a useful tool for plotting GO annotation results, was used to represent GO categories. According to GO distribution out of total count of 32386, molecular function comprised of 24864, followed by biological processes of 21484 and cellular component being 17173. I is worth noting that among the biological processes, metabolic and cellular processes showed the maximum activity.

**Table 8:** Data showing Top 10 Pfam domains identified from RNA-seq of *Z. armatum*.

*Pfam domain*	Count
*Pkinase*	1189
*Pkinase_Tyr*	998
*RRM_1*	464
*p450*	345
*PPR_2*	336
*zf-RING_2*	306
*PP2C*	252
*Sugar_tr*	230
*Ras*	201
*WRKY*	200

**Table 9:** GO Distribution of transcripts obtained from RNA-seq of *Z. armatum*.

Description	Count
*Total GO*	32386
*Biological Process*	21484
*Cellular Component*	17173
*Molecular Function*	24864

Ortholog assignment and mapping of CDS to biological pathways were performed using the KEGG automatic annotation server (KAAS). All CDS were compared against the KEGG database using BLASTP, with a threshold bit-score value of 60 (default). The CDS represents metabolic pathways of major biomolecules such as carbohydrates, lipids, amino acids, nucleotides, glycans, cofactors, vitamins, terpenoids, and polyketides. The mapped transcripts also represented genes involved in metabolism, genetic information processing, environmental information processing, cellular processes and organismal systems (**Supplementary Figure 11**; **Table 10**).

**Table 10:** Statistical data of KEGG pathway analysis for CDS obtained from RNA-seq of *Z. armatum*.

KEGG pathway	Count
*09100 Metabolism*	6407
*09101 Carbohydrate metabolism*	1540
*09102 Energy metabolism*	677
*09103 Lipid metabolism*	796
*09104 Nucleotide metabolism*	277
*09105 Amino acid metabolism*	1001
*09106 Metabolism of other amino acids*	296
*09107 Glycan biosynthesis and metabolism*	353
*09108 Metabolism of cofactors and vitamins*	499
*09109 Metabolism of terpenoids and polyketides*	256
*09110 Biosynthesis of other secondary metabolites*	422
*09111 Xenobiotics biodegradation and metabolism*	290
*09120 Genetic Information Processing*	3279
*09121 Transcription*	508
*09122 Translation*	1230
*09123 Folding, sorting and degradation*	1058
*09124 Replication and repair*	483
*09130 Environmental Information Processing*	2795
*09131 Membrane transport*	50
*09132 Signal transduction*	2744
*09133 Signaling molecules and interaction*	1
*09140 Cellular Processes*	2725
*09141 Transport and catabolism*	1189
*09142 Cell motility*	102
*09143 Cell growth and death*	1068
*09144 Cellular community – eukaryotes*	245
*09145 Cellular community – prokaryotes*	121
*09150 Organismal Systems*	1086
*09149 Ageing*	255
*09158 Development and regeneration*	209
*09159 Environmental adaptation*	622
*09180 Brite Hierarchies*	14001
*09181 Protein families: metabolism*	2343
*09182 Protein families: genetic information processing*	9094
*09183 Protein families: signaling and cellular processes*	2564
*09190 Not included in Pathway or Brite*	744
*09191 Unclassified: metabolism*	551
*09192 Unclassified: genetic information processing*	28
*09193 Unclassified: signaling and cellular processing*	90
*09194 Poorly characterized*	75

A total of 394 pathway maps were found 53 of which were related to biosynthesis (**Table 10**).

Transcriptome data analysis identified putative biosynthetic pathway genes. The present study followed the biosynthetic pathway described in the pathway databases MetaCyc and KEGG (Kyoto Encyclopedia of Genes and Genomes) for the study of berberine and sanguinarine biosynthesis following the EC number (Enzyme Commission number) for enzyme identification.

**Figure 2:**
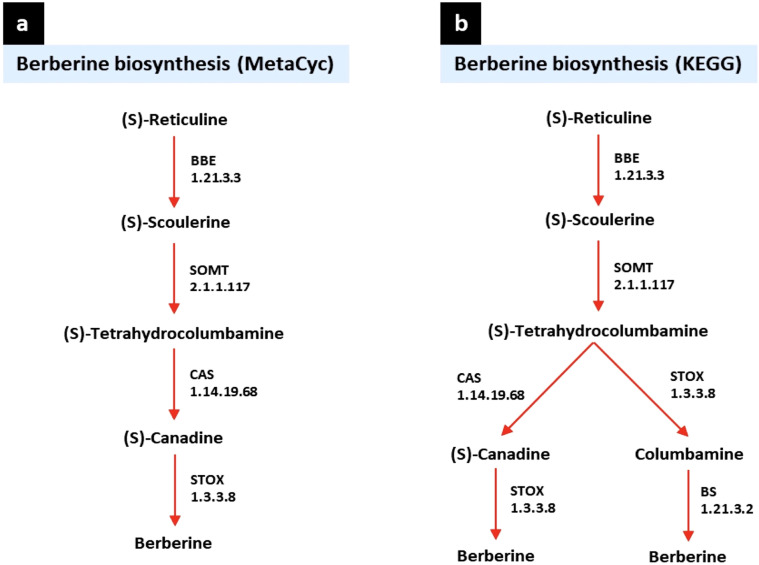
Biosynthetic pathway of berberine including the EC numbers provided in the pathway database **(a)** MetaCyc and **(b)** KEGG.

**Figure 3:**
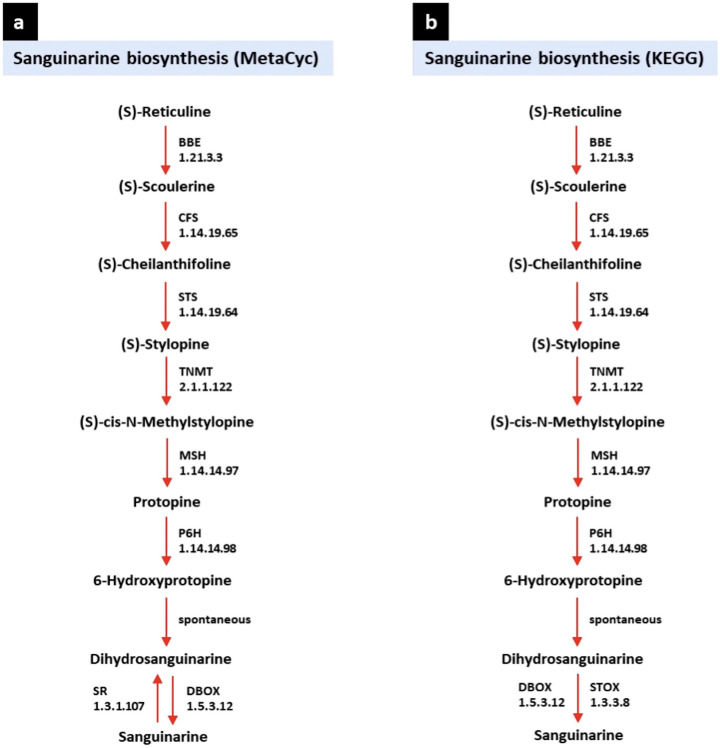
Biosynthetic pathway of sanguinarine including the EC numbers provided in the pathway database **(a)** MetaCyc and **(b)** KEGG.

The enzymes involved leading to the biosynthesis of the two alkaloids berberine and sanguinarine are berberine bridge enzyme (*BBE*), EC 1.21.3.3; (S)-scoulerine 9-O-methyltransferase (*SOMT*), EC 2.1.1.117, (S)-canadine synthase (*CAS*), EC 1.14.19.68; (S)-tetrahydroprotoberberine oxidase (*STOX*), EC 1.3.3.8; berberine synthase (*BS*), EC 1.21.3.2; (S)-cheilanthifoline synthase (*CFS*), EC 1.14.19.65; (S)-stylopine synthase (*STS*), EC 1.14.19.64; tetrahydroprotoberberine cis-N-methyltransferase (*TNMT*), EC 2.1.1.122; (S)-cis-N-methylstylopine 14-hydroxylase (*MSH*), EC 1.14.14.97; protopine 6-hydroxylase (*P6H*), EC 1.14.14.98; dihydro benzophenanthridine oxidase (*DBOX*), EC 1.5.3.12; and sanguinarine reductase (*SR*), EC 1.3.1.107 (**Figures 2**, **3**).

### SSR identification from CDS

Simple sequence repeats (SSRs) or micro-satellites are tandem repeats of 2–6 bp and are highly polymorphic and are ubiquitously present in all known genomes. SSRs were identified in the CDS sequences of each sample using the MISA perl script. A dinucleotide pattern appears at least six times with a trinucleotide pattern five times, tetranucleotide pattern five times, pentanucleotide pattern five times, and hexanucleotide pattern five times (**Tables 11**–**13**). SSRs flanking 150 bp (upstream and downstream) were identified using an in-house python script that can be used for primer design.

**Table 11:** Statistical data after identification of SSRs from the transcript library of *Z. armatum*.

Description	Count
*Total number of sequences examined*	79649
*Total size of examined sequences (bp)*	70755312
*Total number of identified SSRs*	5576
*Number of SSR containing sequences*	4793
*Number of sequences containing more than 1 SSR*	646
*Number of SSRs present in compound formation*	443

**Table 12:** Data showing Pattern Distribution of SSRs from the transcript library of *Z. armatum*.

Unit size	Count
*Dinucleotide*	734
*Trinucleotide*	4606
*Tetranucleotide*	20
*Pentanucleotide*	16
*Hexanucleotide*	200

**Table 13:** Statistics of SSRs with flanking 150 bp regions which are useful for designing of primers needed for molecular marker analysis.

Description	Number of transcripts
*Total number of identified SSRs*	4793
*Number of SSRs with 150 bp flanking region*	1877

It is worth noting that out of the 4793 sequences containing SSRs, 646 sequences contained more than one SSRs and 443 SSRs were in compound form. Trinucleotide repeats were maximum with 4606 sequences, whereas tetra- and pentanucleotide repeats were minimum in number (20 and 16 respectively) which is usually observed in a lot of other plants.

### Production of pathway marker compounds

**Figure 4:**
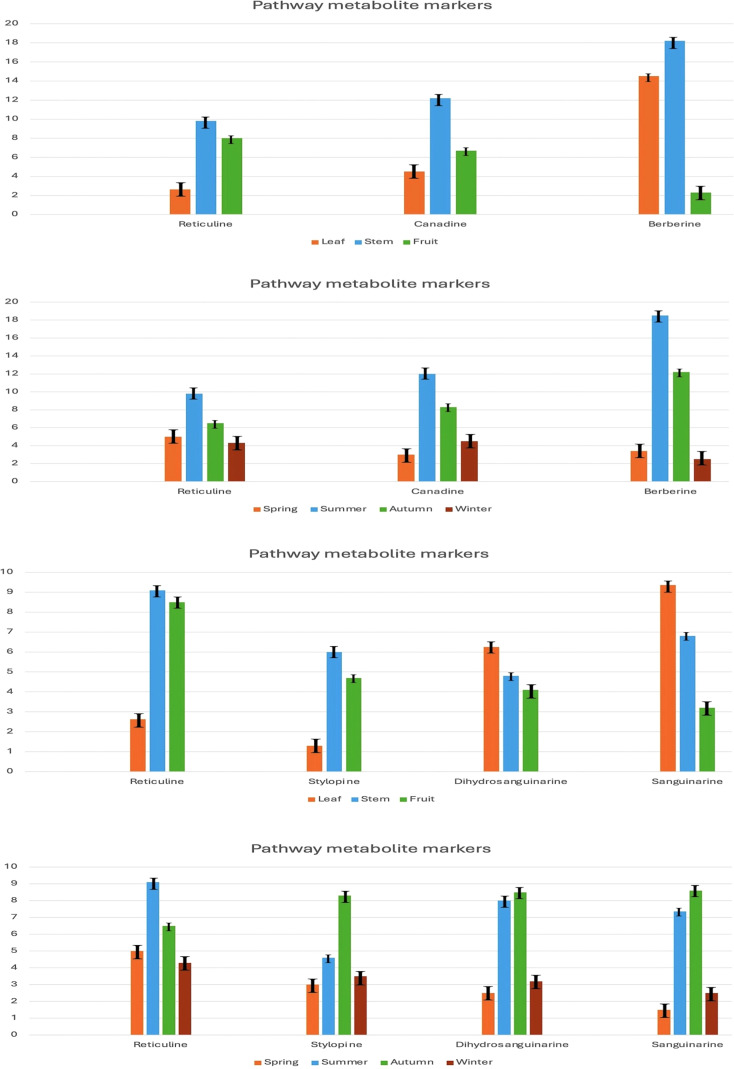
Detection of pathway metabolite markers leading to formation of berberine and sanguinarine in *Zanthoxylum armatum*. Variation in production of reticuline, canadine and berberine is shown in different tissue samples and due to change in season. Variation in production of reticuline, stylopine, dihydrosanguinarine and sanguinarine is shown in different tissue samples and due to changes in season. All data are in mg/g DW (±S.E) with 3 to 4 replicates.

The quantity of berberine in the samples was found to be 14.10±0.25 mg/gDW for leaf, 19.67±1.25 mg/gDW for stem and 2.26±1.41 mg/gDW for fruit. The quantity of sanguinarine in the samples was found to be 9.01±1.12 mg/gDW for leaf, 6.85±1.25 mg/gDW for stem and 3.12±1.2 mg/gDW for fruit (**Figure 4**) (Devi, 2025).

Considering seasonal variation, the highest quantity of berberine in spring was 3.25±0.72 mg/gDW, 18.16±1.22 mg/gDW during summer, 12.22±1.10 mg/gDW during autumn and 2.23±0.65 mg/gDW during winter (**Figure 4**) (Devi, 2025). Production of sanguinarine was 1.50±0.50 mg/gDW during spring, 7.65±1.25 mg/gDW during summer, 8.5±0.75 mg/gDW during autumn and 2.85±0.5 mg/gDW during winter season (**Figure 4**) (Devi, 2025).

Intermediate marker compounds, such as reticuline and canadine, were also estimated using liquid chromatographic analysis. Variations due to season and sample type were also noted and were statistically significant (**Figure 4**). The production of reticuline is highest in the stem, followed by fruit, but is minimal in the leaf. Canadine was produced similarly in stems, followed by fruit, but berberine was produced more in stems and leaves and was significantly less in fruit. When we consider the seasonal variation in production, reticuline production was highest in the summer months and decreased during winter. A similar observation was recorded for canadine production, however, in the case of berberine, production was significantly higher in summer and reduced by nearly ten times in winter. Overall, a very similar trend was observed in the production of all three compounds owing to seasonal variation (**Figure 4**).

When we analyzed the production of intermediate markers leading to sanguinarine formation, a somewhat different picture emerged. Reticuline production was highest in the stem, followed closely by production in fruit. A similar observation was made for the production of stylopine, an important intermediate compound. Dihydrosanguinarine was produced more in leaves, than in stems or fruits, whereas sanguinarine production was significantly higher in leaf samples than in all other compounds (**Figure 4**).

### Differential expression analysis of DEGs

**Figure 5:**
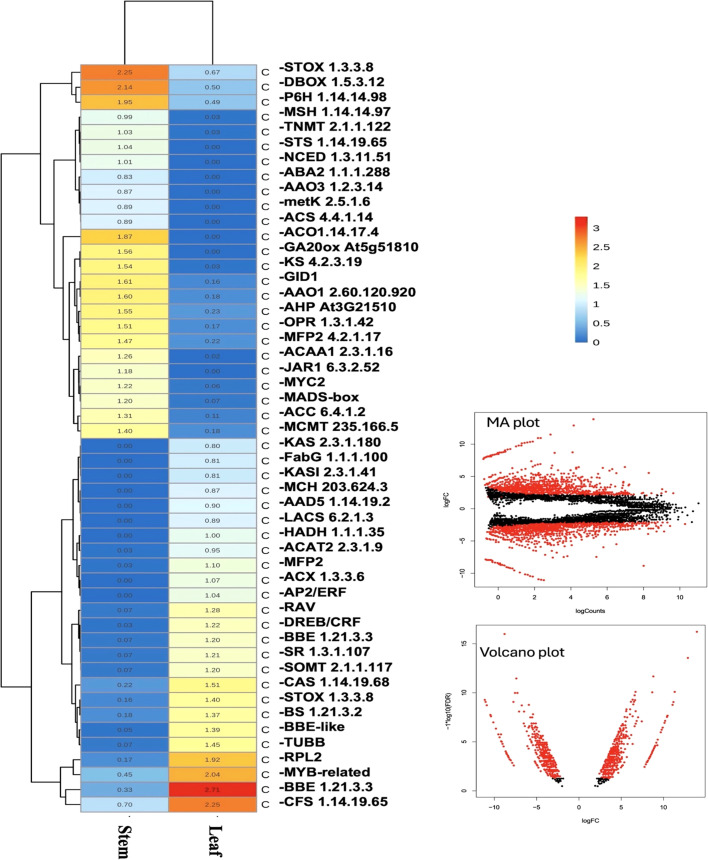
Heatmap representing most significant genes expressed in both the samples was plotted using log10 of normalized read count values (CPM) for Leaf vs Stem, where shades of blue represent downregulated genes and shades of red represents highly expressed genes. MA plot showing differentially expressed transcripts in Leaf vs Stem combination. On X-axis normalized counts for all the samples and on Y-axis log2foldchange are plotted. Points coloured are with red if the adjusted q-value is less than 0.05 and black if the adjusted q-value is greater than 0.05. Volcano plots of the distribution of expressed transcripts in Leaf vs Stem combination. Red corresponds to transcripts with adjusted q-value < 0.05.

**Figure 6:**
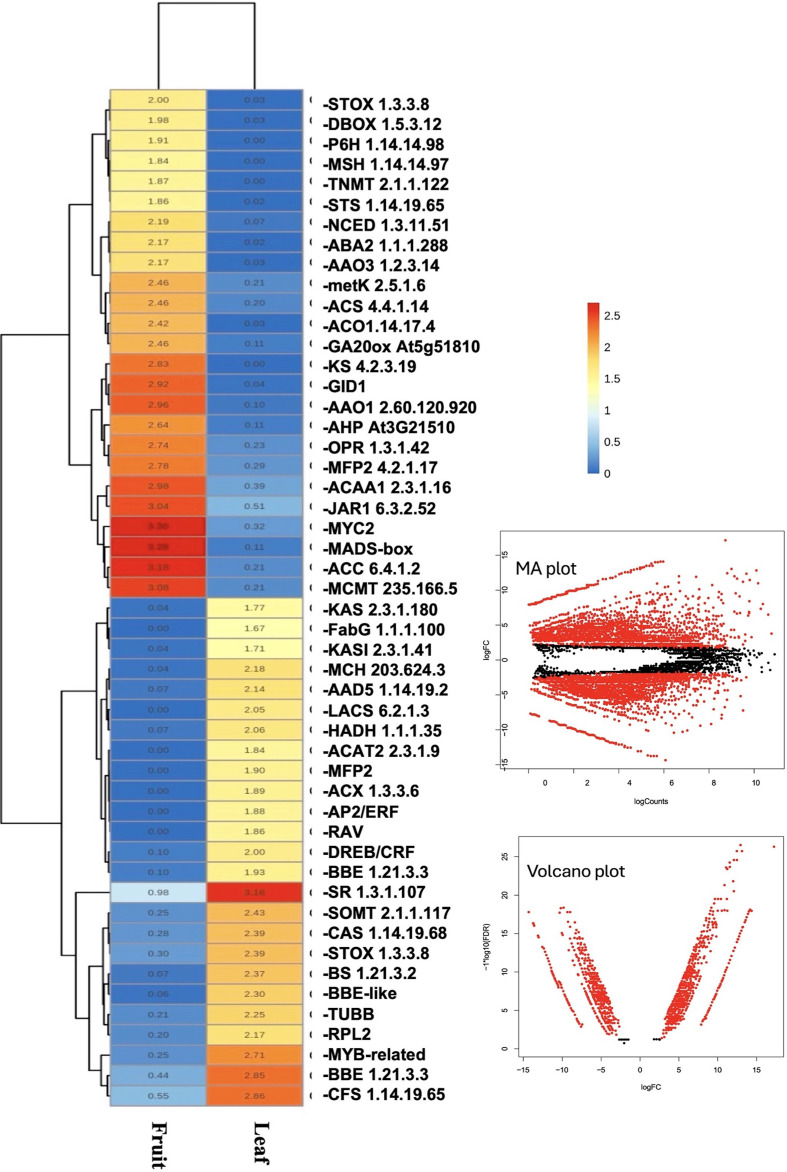
Heatmap representing most significant genes expressed in both the samples was plotted using log10 of normalized read count values (CPM) for Leaf vs Fruit, where shades of blue represent downregulated genes and shades of red represents highly expressed genes. MA plot showing differentially expressed transcripts in Leaf vs Fruit combination. On X-axis normalized counts for all the samples and on Y-axis log2foldchange are plotted. Points coloured are with red if the adjusted q-value is less than 0.05 and black if the adjusted q-value is greater than 0.05. Volcano plots of the distribution of expressed transcripts in Leaf vs Fruit combination. Red corresponds to transcripts with adjusted q-value < 0.05.

**Figure 7:**
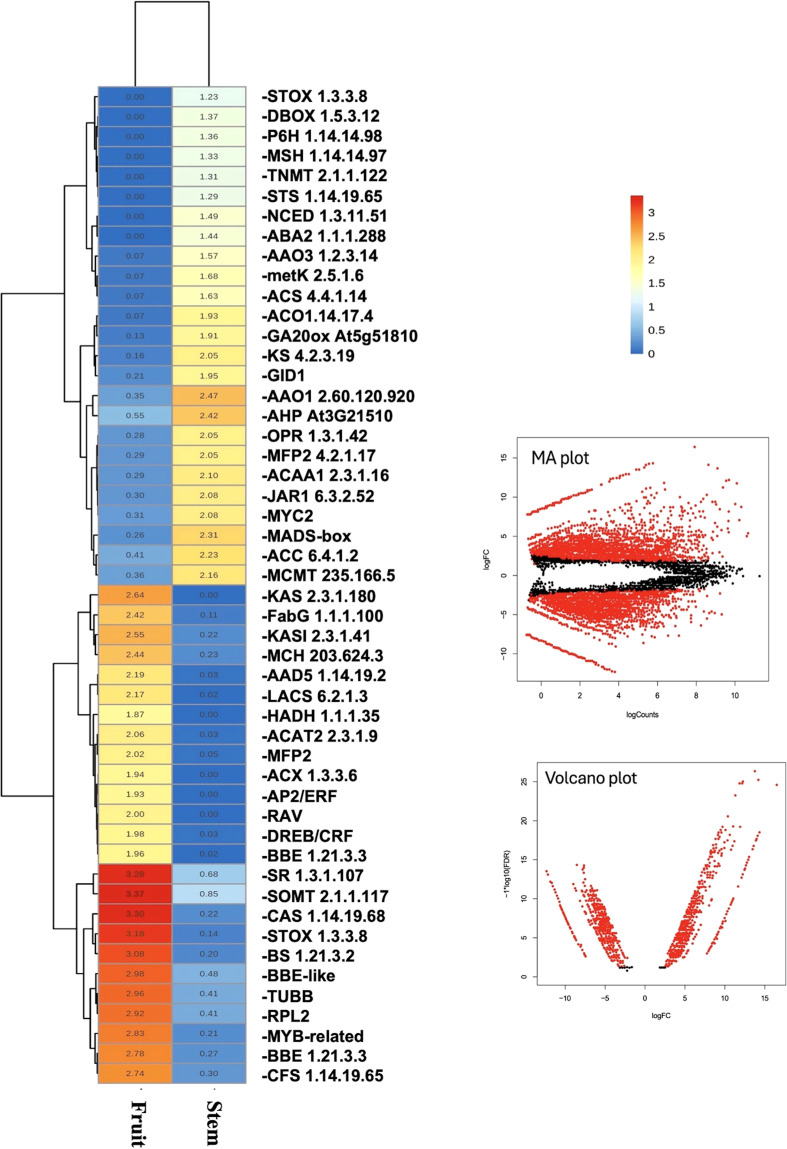
Heatmap representing most significant genes expressed in both the samples was plotted using log10 of normalized read count values (CPM) for Stem vs Fruit, where shades of blue represent downregulated genes and shades of red represents highly expressed genes. MA plot showing differentially expressed transcripts in Stem vs Fruit combination. On X-axis normalized counts for all the samples and on Y-axis log2foldchange are plotted. Points coloured are with red if the adjusted q-value is less than 0.05 and black if the adjusted q-value is greater than 0.05. Volcano plots of the distribution of expressed transcripts in Stem vs Fruit combination. Red corresponds to transcripts with adjusted q-value < 0.05.

The combinations considered for the DEG analysis were leaf Vs stem, leaf Vs fruit, and stem Vs fruit. Steps performed to obtain DEGs were as follows: reads of each sample were mapped separately to CDS sequences obtained from master assembly using bwa (Version: 0.7.12-r1039); duplicate PCR reads were removed from the alignment file using Picard’s MarkDuplicates.jar; Finally, these mapped reads in the above-mentioned combination were considered for DEG analysis and the read mapped count from each sample was given as an input for differential expression analysis using the edgeR bioconductor package in R. Differential gene expression was inferred between sample groups by applying the R package edgeR (v3.28.1). edgeR is a bioconductor package based on the negative binomial distribution method. Differential expression analysis statistics for the combinations of leaf vs stem, leaf vs fruit, stem vs fruit are provided in **Tables 14**, **15** and **Figures 5**–**7**. Genes that were also considered were phytohormone synthesis and fatty acid synthesis related (**Supplementary Figures 13**–**15**).

**Table 14:** Criteria used to identify upregulated and down regulated genes/transcripts and assigning the significance.

Condition	Status
*log2FC>0*	Up regulated
*log2FC<0*	Down regulated
*log2FC>0 and q-value >0.05*	Significantly up regulated
*log2FC<0 and q-value <0.05*	Significantly down regulated

**Table 15:** Data showing Differential Gene Expression from various samples of *Zanthoxylum armatum*.

Combination	Leaf Vs Stem	Leaf Vs Fruit	Stem Vs Fruit
*Total Differentially Expressed Genes*	62824	62828	62321
*Down - regulated*	31762	32278	31754
*Up – regulated*	31062	30550	30567
*Significantly - Downregulated*	1629	6848	5621
*Significantly - Upregulated*	1309	4657	3791

**Figure 8:**
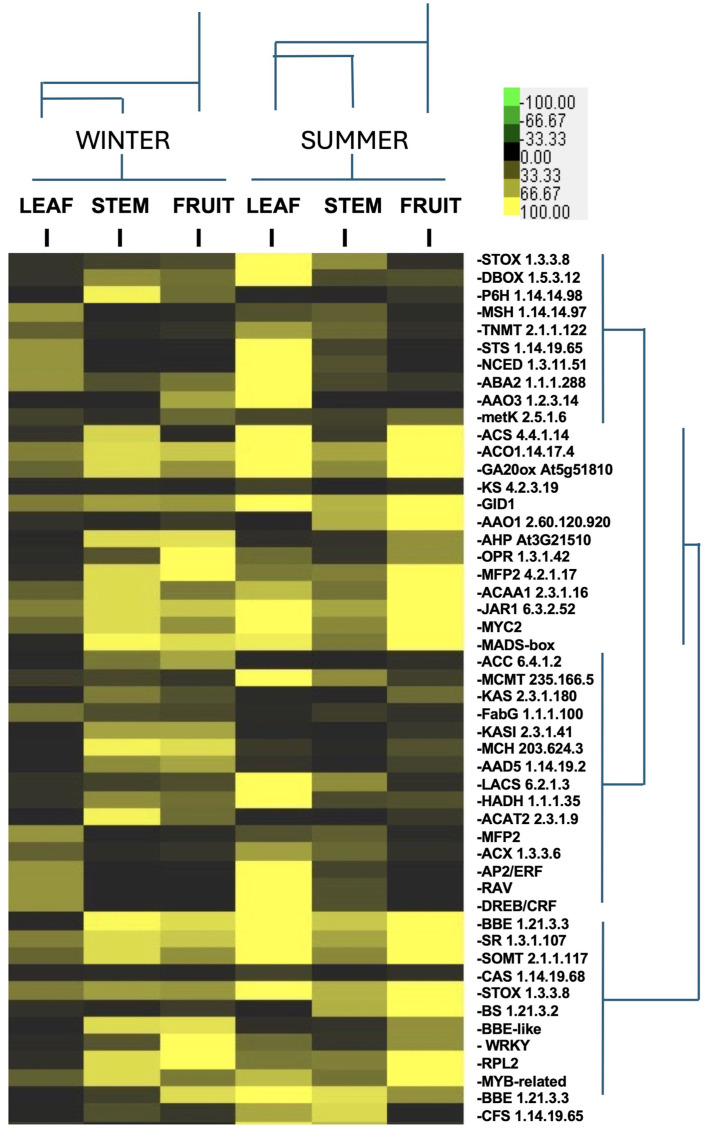
Heatmap of qRT-PCR detected genes/transcripts from stem, leaf and fruit samples of *Zanthoxylum armatum*.

Phytohormones are important for plant growth and development [4]. They may also play a role in alkaloid or fatty acid biosynthesis (**Figure 8**). Among them, we considered 9-cis-epoxycarotenoid dioxygenases (*NCED*), xanthoxin dehydrogenase (*ABA2*), and abscisic-aldehyde oxidase (*AAO3*), which are the key genes involved, in their representation in the library. The transcriptome data indicated many key genes that are variously up- or down-regulated in the different tissue samples and in different seasons. Prominent among them are S-adenosylmethionine synthetase (*metK*), ACC synthases (*ACS*), *ACC oxidases* and ethylene-responsive transcription factor (*ERF*) which were up-regulated and downregulated in the different samples. DEGs such as gibberellin 20 oxidase (*GA20ox*), ent-kaurene synthase (*KS*), gibberellin receptor (*GID1*), Indole-3-acetaldehyde oxidase (*AAO1*) were up-regulated in fruit samples compared to the other two samples (**Figure 8**). The histidine-containing phosphotransfer peotein (*AHP*) was significantly highly expressed in fruit samples but remained uniform in stem and leaf samples. 12-oxophytodienoic acid reductase (*OPR*), enoyl-CoA hydratase (*MFP2*), and acetyl-CoA acyltransferase 1 (*ACAA1*) were upregulated; jasmonate-amino acid synthetase (*JAR1*) and transcription factor *MYC2* were downregulated in stem samples (**Figure 8**). These results show that genes related to various key pathways may be involved in plant development, during sampling.

Among the pathway genes leading to formation of berberine, genes BBE, SOMT, CAS, STOX, BS and BBE-like all showed upregulation in leaf tissues as compared to stem and fruit samples throughout all the four seasons of the year when samples were collected.

Genes leading to formation of sanguinarine, CFS, SR and MYB-related were upregulated in leaf tissues, but the other genes like TNMT, P6H, MSH, DBOX and STS were upregulated at various degrees in fruit samples which was a new result and was unexpected.

Other genes which were considered for expression analysis al showed overexpression and upregulation in the fruit samples throughout the 4 seasons of the year. These included important genes like NCED, AAO1, AAO3, ACS, ACOAHP, OPR and ACC, genes which play important roles as oxidoreductases.

KEGG analysis revealed that, these DEGs were associated with subfamilies, including ACP desaturase 5 (*AAD5*), ketoacyl-ACP synthetase I (*KASI*), long-chain acyl-CoA synthase (*LACS*), ketoacyl-CoA synthase (*KCS*), and oxoacyl-acyl-carrier protein reductase (*FabG*), for which homologous sequences were not identified in the TAIR10 protein database. Additionally, *KAS*, *FabG*, *AAD5*, *LACS* and *KCS* were mainly associated with fatty acid biosynthetic processes.

Furthermore, *KAS* and *KASI* were significantly upregulated in fruits during winter (**Figure 8**). However, *FabG*, which reduces3-Oxo-N-ACP to form 3-hydroxy-N-ACP, was upregulated to a lesser extent in fruits. It is worth noting that three unigenes annotated to *AAD5* showed similar patterns and were exclusively upregulated in fruits throughout all seasons.

The major pathways of fatty acid degradation were beta-oxidation catabolism, and some of the enzymes involved in this step were identified in our DEG profiles. These included long-chain acyl-CoA synthase (*LACS*), acyl-CoA oxidase (*ACX*), multifunctional protein 2 (*MFP2*), hydroxyacyl-CoA dehydrogenase (*HADH*), and acetoacetyl-CoA thiolase 2 (*ACAT2*) which were represented in various samples and in the assembled transcriptome library (**Figure 8**).

### Identification of transcription factor

**Figure 9:**
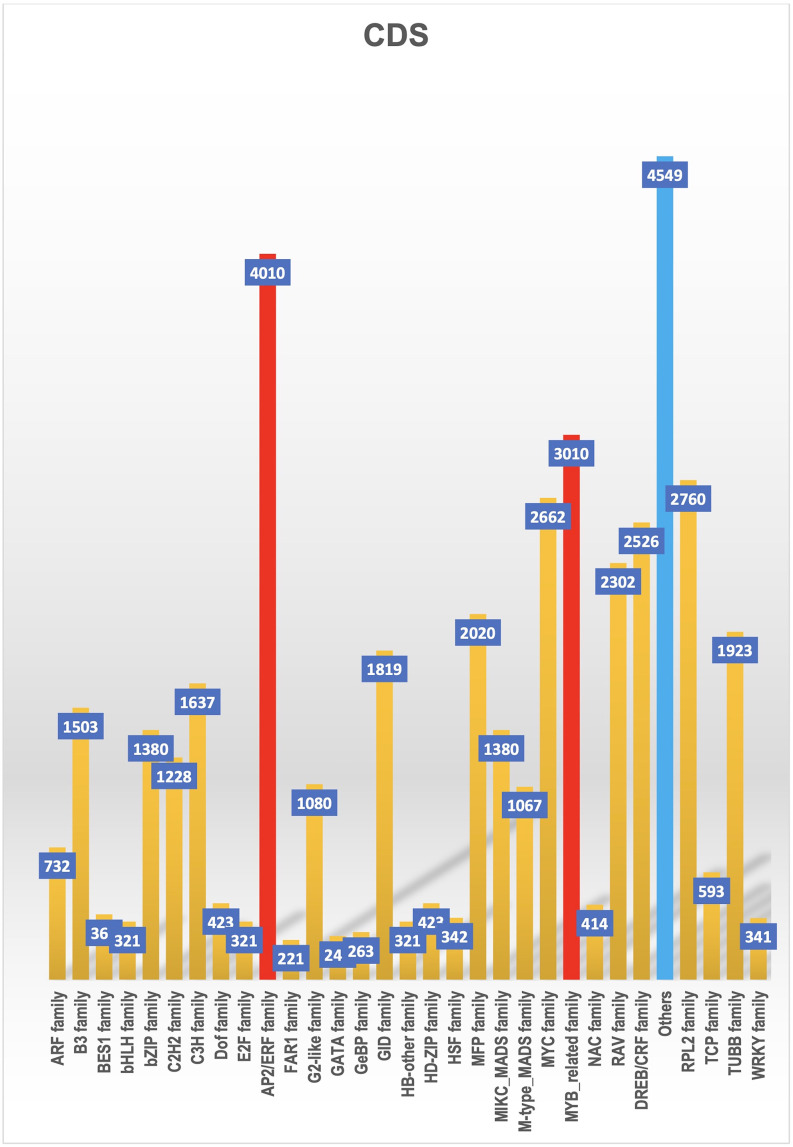
Transcription factor family distribution, AP2/ERF family and MYB-related family arehighlighted with red and Others group is highlighted with blue.

**Figure 10:**
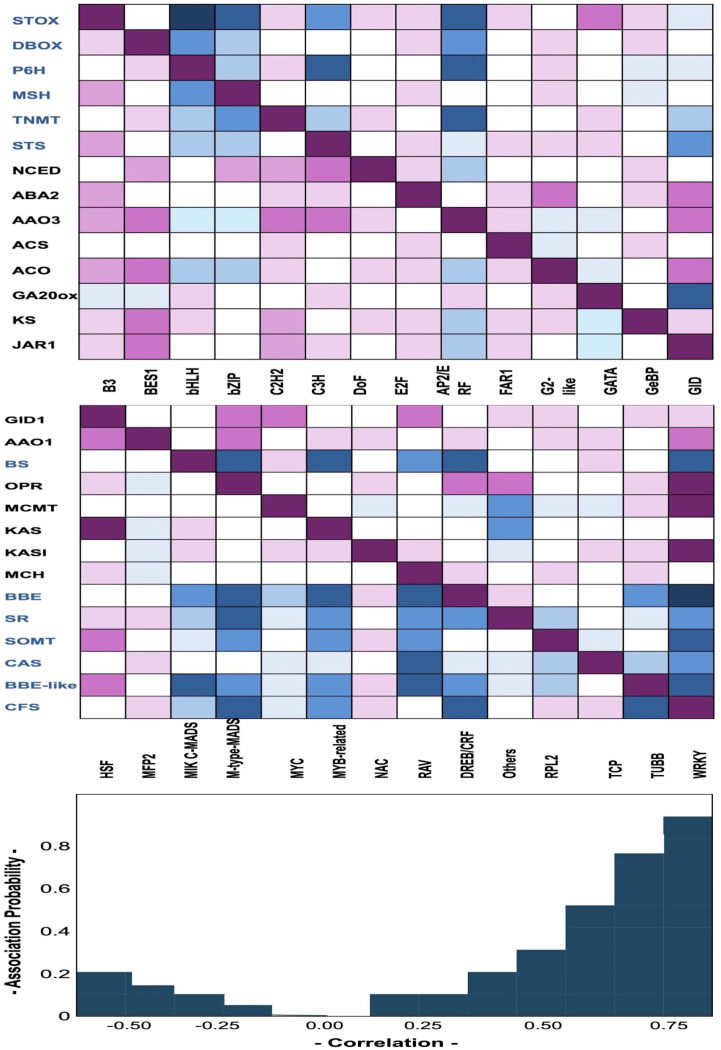
Correlation matrix for genes and transcription factors (TFs) that were detected and represented in numbers in the transcriptome of *Zanthoxylum armatum*. Genes are ordered by hierarchical clustering to reveal numerous modules of highly correlated and/or anticorrelated genes. The genes in these modules are enriched for specific transcription factors. Genes with known functional roles in the process of alkaloid biosynthesis in *Z armatum* and their regulation by TFs are shown where genes with high correlations with particular TFs are clearly identified by blue as related to genes which have less or no correlation in purple.

**Figure 11:**
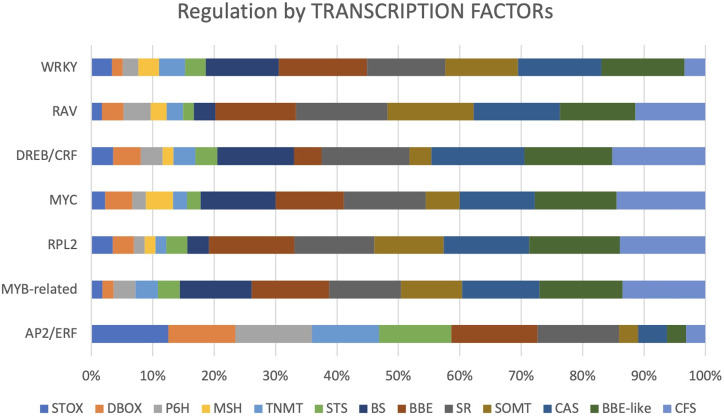
Regulation of important genes from the berberine and sanguinarine biosynthesis pathway by the identified 7 Transcription Factor superfamilies.

In the *Z. armatum* transcriptome data, 42177 CDS encoded transcription factors. Of these, 38808 CDS could be identified and assigned to 64 transcription factor (TF) families comprising various counts. Families having counts (320 and below) are grouped and named as ‘Others’ (**Figure 9**) covering 34 families. The three most abundant TF families were *AP2/ERF* family, *MYB*-related and *RPL2* families. The largest groups of TFs was the *AP2/ERF* family (4010), followed by *MYB-*related family (3010), *RPL2 family* (2760), *MYC family* (2662), *DREB/CRF family* (2526), and *RAV* (2302) families (**Figures 10**, **11**). These results also included factors, such as *WRKY*, *MFP*, *TUBB*, *GID*, *C3H*, and *bZIP* zinc finger proteins, which were also expressed.

### qRT-PCR analysis

The selected primers produced the expected amplicon size in semi-quantitative PCR, indicating the presence of the genes under study in the tested samples (**Figure 8**). qRT-PCR was performed for 50 selected DEGs, and six DEGs namely *BBE-like*, *TUBB*, *RPL2*, *MYB-related*, *BBE*, and *SR*, confirmed the reliability of transcriptome data. Linear regression analysis of the relationship between differential expression given by bioinformatic predictions (log2FC by WTS) and qRT-PCR analysis (log2FC by RT-qPCR) showed a high correlation between them with a coefficient of determination, R2 = 0.8154 (**Figure 8**) indicating that the transcriptome data were reliable.

The putative biosynthetic pathway gene(s) of berberine (*BBE*) and sanguinarine (*BBE* and *SR*) identified in the transcriptome data were found to be expressed in qRT-PCR experiments in all tested samples of plant 7 collected during the flowering and fruiting stages of the plant (**Supplementary Figure 12**).

Genes such as *BBE* were found to be expressed in all tested samples of *Z. armatum*. The relative expression was found to be higher in leaves in all the studied stages except during winter months when the expression was similar. For the *SR* gene, the relative expression was found to be higher in leaves, in all the stages under study, except in the winter months when it was similar in all samples. The genes were expressed at higher levels in leaves than in stems and at higher levels in stems than in fruits in all fruiting stages (**Figure 8**).

To demonstrate the correlation between the pathway genes and TFs, it was necessary to additionally work on the modules generated for genes and TFs so that a direct correlation can be visualized, rather than considering samples and seasonal variations. For this analysis, we considered 28 genes and 28 TFs in the correlation matrix module (**Figures 9**, **10**). TFs *bHLH* and *bZIP* were positively correlated with genes such as *STOX*, *DBOX*, *P6H*, *MSH*, *TNMT* and *STS*, and partially positively correlated with *AAO3* and *ACO*. *C3H* levels were positively correlated with *STOX*, *P6H* and *TNMT*. *AP2/ERF* positively correlated with *STOX*, *DBOX*, *P6H*, *TNMT*, *STS*, *NCED*, and was partially positive for *ACO*, *KS* and *JAR1*. *GID* family was positively correlated with *STOX*, *P6H*, *TNMT*, *STS* and *GA20x* expression (**Figure 10**).

The TFs *MIKC-MADS* and *M-type-MADS* were positively correlated with genes such as *BBE*, *SR*, *SOMT*, *CAS*, *BBE-like* and *CFS*. The TFs *RAV* and *DREB/CRF* were positively correlated with *BBE*, *SR*, *SOMT*, *CAS*, *BBE-like* and *CFS*. TF *MYB-related* were positively correlated with *BS*, *BBE*, *SR*, *SOMT*, *CAS*, *BBE-like* and *CFS*. *WRKY* was positively correlated with *BS*, *BBE*, *SR*, *SOMT*, *CAS* and *BBE-like* (**Figures 10**, **11**).

## Summary of results

A total of 6.7, 7.0 and 5.9 GB HQ data of fruit, leaf, and stem samples were used for *de novo* assembly using Trinity. Assembled transcripts were clustered using CD-hit software resulting into 44254, 46402 and 46521 unigenes in leaf, fruit, and stem samples, respectively.

A total of 27777, 32118 and 19754 CDS in fruit, leaf and stem samples, respectively, were predicted from unigenes using Transdecoder software. The protein sequences of leaf, fruit and stem samples were searched for similarity against the NR, UniProt, KOG and Pfam databases. The GO distribution was determined using Blast2GO Pro. In fruit samples, 4802 CDS were assigned to Biological Process, 5453 CDS to Cellular Component and 2321 CDS to Molecular Function, whereas in leaf samples, 3927 CDS were assigned to Biological Process, 4211 CDS to Cellular Component and 2101 CDS to Molecular Function. In the stem sample, 3452 CDS were assigned to Biological Processes, 3622 CDS to Cellular Components and 2497 CDS to Molecular Function. The pathway analysis was performed, using the KAAS server. The CDS were enriched and predominantly categorized into Metabolism, Genetic Information Processing, Environmental Information Processing and Cellular Processes. A total of 2744 CDS contributed to the activity of the Signal Transduction pathway in the three sample types and 6407 CDS contributed to Metabolism. SSR identification using MISA resulted in 5576 SSRs identified from fruit, leaf and stem samples, out of which 1877 SSRs with 150 flanking regions were predicted.

Principal Component Analysis (PCA) associates’ transcripts related to detectable intermediates and final product formation, for both berberine and sanguinarine. It is a statistical methodology used to statistically prove the significance of metabolically synthesized products and the genes involved in their synthesis, and conclusively show their relatedness (**Supplementary Figure 16**).

## Discussion

Biochemical and molecular studies of *Zanthoxylum armatum* DC. has a future scope for further exploration of its medicinal properties, particularly its potential as an anticancer agent, and developing sustainable cultivation and harvesting methods. Gene expression studies provide insights into the cellular effects, and a multi-omics approach integrating transcriptome, metabolome and proteome analyses can reveal various molecular mechanisms of *Z. armatum* DC.

### Analysis of transcriptome

The present study involved *de novo* transcriptome assembly and analysis of leaves, stems and fruits of *Zanthoxylum armatum* DC. predicted 137177 unigenes from the master assembly of 164899 transcripts and 79649 CDS were predicted from the unigenes. The similarity search for functional annotation of the protein sequences of the predicted CDS against four databases using BLASTP resulted in annotation of 74049 (92.97%) CDS in NR, 60331 (75.75%) CDS in UniProt, 38489 (48.32%) CDS in KOG and 38111 (47.85%) CDS in Pfam. A total of 6.7, 7.0 and 5.9 GB HQ data of fruit, leaf and stem samples were used for *de novo* assembly using Trinity. Assembled transcripts clustered using CD-hit software resulted in 44254, 46402 and 46521 unigenes in leaf, fruit, and stem samples respectively (Devi, 2025).

A total of 27777, 32118 and 19754 CDS in fruit, leaf, and stem samples, respectively, were predicted from unigenes using Transdecoder software. The protein sequences of the leaf, fruit, and stem samples were searched for similarity against the NR, UniProt, KOG, and Pfam databases. The GO distribution was determined using Blast2GO Pro. In fruit samples, 4802 CDS were assigned to Biological Process, 5453 CDS to Cellular Component and 2321 CDS to Molecular Function whereas in leaf samples, 3927 CDS were assigned to Biological Process, 4211 CDS to Cellular Component, and 2101 CDS to Molecular Function. In the stem sample, 3452 CDS were assigned to Biological Processes, 3622 CDS to Cellular Components and 2497 CDS to Molecular Function categories.

Annotation analysis identified 17 CDS from 17 unigenes for the putative biosynthetic pathway gene, berberine bridge enzyme, and two CDS from two unigenes for the putative biosynthetic pathway gene, sanguinarine reductase. The expression of these putative biosynthetic pathway gene(s) was analyzed using semi-quantitative PCR and qRT-PCR and different expression levels (fold-change) were observed by qRT-PCR. In the present study, berberine and sanguinarine were part of the pathway map ko00950 (isoquinoline alkaloid biosynthesis) (Devi, 2025).

An earlier comparative transcriptome analysis of the male and female flowers of *Zanthoxylum armatum* var. *novemfolius* obtained a total of 50605 unigenes and annotated 48104 unigenes in NR, 36542 unigenes in Swiss-Prot, 29492 in Pfam, 8348 unigenes in COG, 27148 unigenes in GO and 20362 unigenes in KEGG databases (Zhang et al., 2020). Species distribution of the BLAST of transcriptome resulted in closely matched with the sequences of *Citrus sinensis* (43.68%), followed by *Citrus clementina* (31.76%), *Vitis vinifera* (2.05%) and *Theobroma cacao* (1.32%) (Hui et al., 2020). Similarity with *Citrus* species has also been observed by Devi and Das (2022), perhaps because of the availability of annotated complete genome assembly in the public domain at the time of analysis.

*De novo* transcriptome analysis of stem tips of *Z*. *bungeanum*, a total of 45,057 unigenes and 22,212 protein coding sequences were predicted, approximately 90% of which showed significant similarities to known proteins in databases (Guo et al, 2012). To identify candidate genes related to sanshool biosynthesis in *Zanthoxylum bungeanum*, the transcriptomes of pericarps from three developmental stages were comparatively analyzed. Differentially expressed gene (DEG) analysis revealed that 2322 unigenes were significantly upregulated or downregulated, significantly among the three types of pericarps and 176 of the DEGs were putative transcription factors (TFs) belonging to 40 TF families (Beaudoin and Facchini, 2013).

*De novo* transcriptome analysis of the stem tips of *Z*. *bungeanum* revealed a total of 45,057 unigenes, and 22,212 protein-coding sequences showed significant similarities to known proteins in databases (Guo et al., 2012; Feng et al., 2017; Li et al., 2019). In a study on seasonal transcriptome profiling of susceptible and tolerant *Citrus* cultivars, RNA-seq analyses of susceptible and tolerant varieties during winter, spring, summer, and fall revealed significant variations in DEGs. The highest number of DEGs was observed in spring for both varieties (Li et al., 2019). Seasonal variation was also observed in the transcriptomic profiling of *Tetrastigma hemsleyanum* fully developed tuberous roots (Ribeiro et al., 2023).

### Identification and analysis of genes

Chung et al. (2006) investigated the role of hormones during flower development. Sixteen key genes belong to the ethylene pathway, including *met*K, *ACS* and *ERF*. Seventeen DEGs from the gibberellin biosynthesis pathway and ten DEGs associated with the gibberellin signal transduction pathway, with *gibberellin 20 oxidase (GA20ox)* having a higher expression in fruits than in leaves in *Z. armatum*. However, *ent-kaurene synthase* (*KS*) and *gibberellin receptor* (*GID1*) showed higher expression in fruits, than in stems across seasonal variations. Indole-3-acetaldehyde oxidase (*AAO1*) was steadily up-regulated from leaves to fruits. From the *Z. armatum* transcriptome data, *histidine-containing phosphotransfer peotein* (*AHP*) in the cytokinin pathway was expressed significantly higher in fruit across the seasonal variations. *12-oxophytodienoic acid reductase* (*OPR*), enoyl-CoA hydratase (*MFP2*), and *acetyl-CoA acyltransferase 1* (*ACAA1*) were significantly up-regulated in fruits across the seasons; *Jasmonate-amino acid synthetase* (*JAR1*) and *transcription factor MYC2*, involved in the jasmonic acid signal transduction pathway, were downregulated in leaf samples. These results indicated that genes related to plant hormones may play a role in alkaloids biosynthesis in *Z. armatum*.

*BBE* is a branch point enzyme in benzylisoquinoline alkaloid production (Hajra et al., 1997) and was also isolated from cell cultures of *Macleaya macrocarpa* (Kutchan, 1998). BBE activity has also been reported in plant cell cultures of various species belonging to the Papaveraceae and Fumariaceae families (Rink and Böhm, 1975) and was expressed heterologously in insect cell cultures (Steffens et al., 1985; Kutchan and Dittrich, 1995). This enzyme was also reported in the methylotrophic yeast *Komagataella phaffii* (Kutchan et al., 1994). Winkler et al. (2008) elucidated the crystal structure and Kutchan et al. (1994) have investigated the catalytic mechanism of this enzyme. The genes of *BBE*-like enzymes form a multigene family but most of them do not carry out alkaloid biosynthesis proving that *BBE*-like enzymes may play a general role in plant development virtually. The question of the general function(s) of such enzymes in plant physiological activities is connected to reactions in primary metabolism inevitably catalyzed by members of their own family.

The stabilized increase in the *BBE*-enzyme family in bacteria, fungi and particularly in plants clearly proves their evolutionary success and will be interesting to study such enzymes further.

The effect of berberine from *Zanthoxylum armatum* has also been reported in previous studies (Ober and Kaltenegger, 2009; Zhou et al., 2019; Jia et al., 2018). However, reports of the gene expression or genomic studies on berberine synthesis are scarce (Yamada et al., 2015). *STOX*, which catalyzes (S)- canadine to berberine, was purified from cell suspension cultures of *Berberis wilsoniae* (Yamada et al., 2017), and the activity of recombinant *STOX* was demonstrated by heterologous expression (Salmore and Hunter, 2001). Our future efforts will focus on the priming of such gene sequences for the overproduction of these compounds.

Comparative transcriptome analysis for the formation of benzylisoquinoline alkaloids (BIAs) and bis-benzylisoquinoline alkaloids (bisBIA) was reported earlier, quantified and characterized (Gesell et al., 2011; Bhardwaj and Kaushik, 2013; Mokhber-Dezfuli et al., 2014; Hagel and Facchini, 2013). The berbamine biosynthesis pathway was first elucidated by Roy et al. (2021). Therefore, functional studies of the pharmacological effects of the target compounds in *Zanthoxylum armatum* are required.

Both *Zanthoxylum* and *Berberis* contain bisbenzylisoquinoline (BIAs), with berberine being the major molecule. We identified all genes encoding berberine synthesis in the transcriptomes of the three sample types but berbamine could not be detected in the biochemical analysis in our study. Comparative genomic studies have not been performed on the transcriptomes of different species of *Zanthoxylum* as *Z. armatum* is the only species available abundantly in India. Thus, future studies should focus on other species for berbamine production.

The berberine bridge enzyme (*BBE*) converts (S)-reticuline to (S)-scoulerine, with the initial conversion leading to sanguinarine pathway (Gesell et al., 2011) using an alternative pathway proposed by Bhardwaj and Kaushik (2013).

Various studies have identified the accumulation of benzylisoquinoline alkaloids (Bhardwaj and Kaushik, 2013; Mokhber-Dezfuli et al., 2014; Hagel and Facchini, 2013; Roy et al., 2021; Nguyen and Kou, 2021; Roy et al., 2022; Facchini et al., 1996b; Unterlinner et al., 1999; Facchini et al., 1996b; Bird et al., 2003; Eilert and Constabel, 1985; Widholm, 1972; Laines-Hidalgo et al., 2022). There is hardly any uniform sanguinarine accumulation that could be linked to factors such as the differential expression of these biosynthetic genes due to environmental or other edaphic factors, or the differential expression of transporter protein related to alkaloid secretion, subject to the availability of sanguinarine.

### Analysis of transcription factors

In the correlation matrix obtained, equally positively and negatively correlated measurement noise is expected, considering all genes as independent. The positive pair-wise correlations in our data arose primarily from genes that share common regulatory transcription factors. Genes within a module have positive correlations pairwise and correlations between different modules are often negative because of phase differences. A negative correlation between certain pairs of genes is mechanistically possible, and a more positive correlation indicates that coregulation of a group of genes by a transcription factor is common. In the *Z. armatum* transcriptome data, 42177 CDS-encoded transcription factors were identified. The largest group of TFs was the *AP2/ERF* family (4010), followed by *MYB-*related family (3010), *RPL2 family* (2760), *MYC family* (2662), *DREB/CRF family* (2526), and *RAV* (2302) (**Figure 9**). These results also included factors such as *MFP*, *TUBB*, *GID*, *C3H*, and *bZIP* zinc finger proteins (**Figures 9**–**11**). These results imply that *AP2*/*ERF*, *MYB-related*, *RPL2, MYC, DREB/CRF, RAV* and *WRKY* are TF superfamilies in this plant species. The *AP2*/*ERF* family is also involved in the regulation of plant flower development. In *Zanthoxylum armatum*, we observed its expression in stems and leaves, which has never been observed earlier. These results when compared to the transcriptome data of *Ribes diacanthum*, were similar, where *bZIP*, *MYB*, *WRKY*, *bHLH*, zinc finger, and *PHD* finger proteins were the largest TF groups (Ober and Kaltenegger, 2009; Zhou et al., 2019; Jia et al., 2018) and to another transcriptome data where the largest TF groups were *MYB*, *bHLH*, *NAC*, *AP2*/*ERF*, and *MADS-box* (Ober and Kaltenegger, 2009).

The heterologous expression of *WRKY* protein in *E. californica*-cultured cells increased the content of sanguinarine along with other components concomitant to an increase in expression of biosynthetic genes (Laines-Hidalgo et al., 2022; He et al., 2018; Han et al., 2016; Singh and Sharma, 2018).

## Conclusion

The putative biosynthetic pathway genes *BBE* (berberine bridge enzyme) of the berberine and sanguinarine pathway and SR (sanguinarine reductase) of sanguinarine pathway were identified and found to be expressed in different parts of the plant in different seasons and at different levels. Transcription factor families such as *AP2/ERF*, *RAV*, *RPL2*, *MYB-related, DREB/CRF, MYC* and *WRKY* involved in biosynthesis were identified, and the correlation matrix showed a positive correlation with the genes of the pathway.

Sequences of the genes of *Z. armatum* DC. obtained in this study provide additional sequence resources that will be beneficial for future research. The increase in research data regarding the phytochemicals and biological activities connecting the traditional uses of the plant further enhances the importance of the plant. This study also provides important information about the target compounds identified from different parts of the plant collected during different seasons and provides a starting point for future breeding and selection for improvement of the plant.

The knowledge generated should be directed towards the generation of tools for commercial exploitation by modern methodologies, such as cell culture and synthetic biological approaches. The availability of the complementary genes for sanguinarine synthesis, along with the regulatory elements and those involved in their mobilization, allows the improvement of enzymatic catalysis by gene editing and the internal cell cascade of intermediaries, resulting in more efficient processes for the accumulation of these alkaloids. Towards that end, our laboratory is presently utilizing nanoparticles and artificial intelligence-based designing of efficient miniPromoters to enhance the production of target compounds in a more efficient and predictable manner.

## Data Availability

The datasets presented in this study can be found in online repositories. The names of the repository/repositories and accession number(s) can be found in the article/**Supplementary Material**.
