# Supplementary material for: Whole transcriptome sequencing from Zanthoxylum armatum: implications on metabolic pathway analysis and regulation
Source: Front Plant Sci. 2026 Jun 11;17:1795497. doi: 10.3389/fpls.2026.1795497 (PMC13294103; doi:10.3389/fpls.2026.1795497)
Supplement: Supplementary file 1 [file DataSheet1.docx]

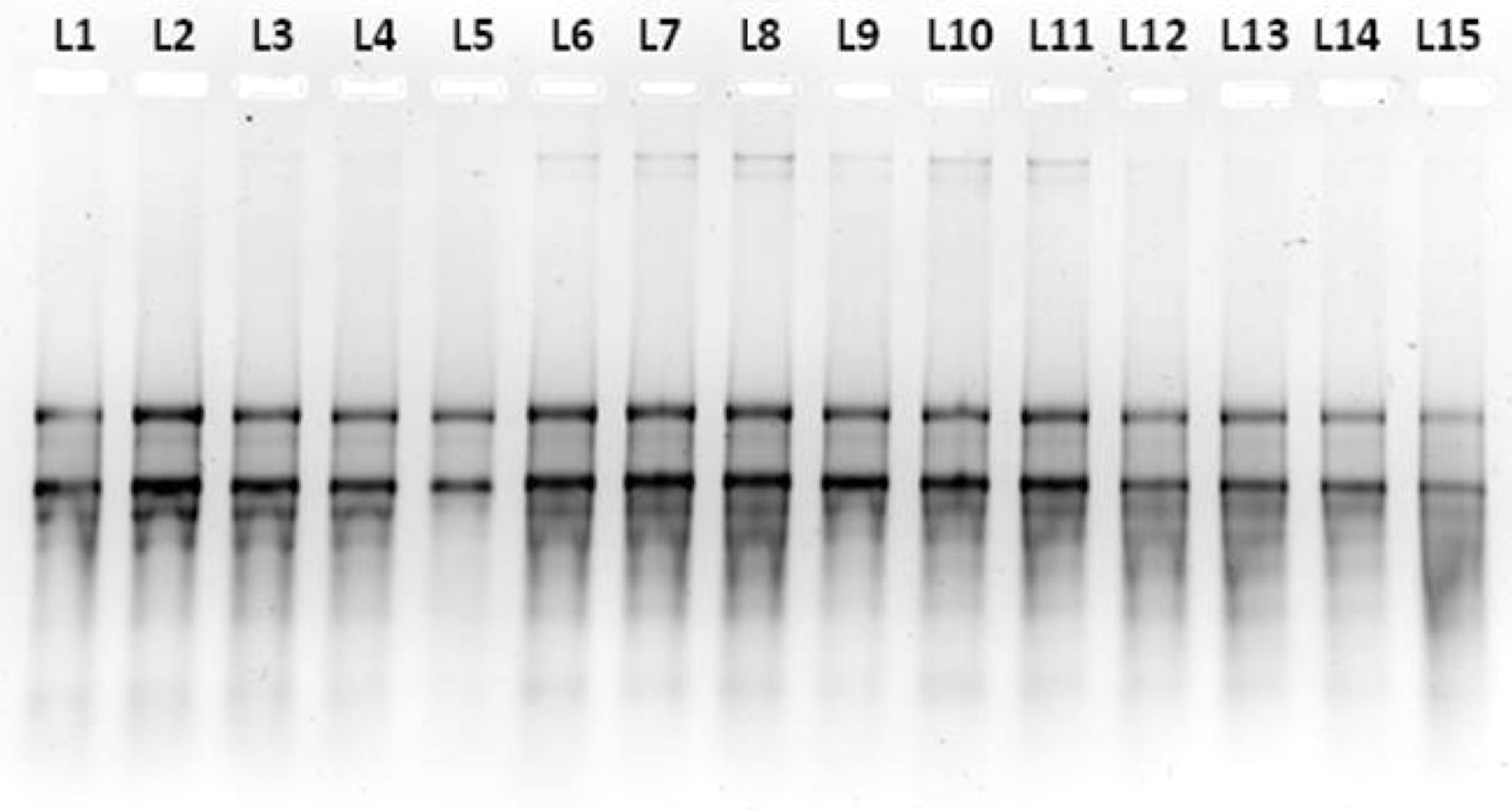


Supplementary Fig. 1. Agarose gel electrophoresis of total RNA isolates before DNase I treatment. L3, L7 and L13 were used for sequencing.


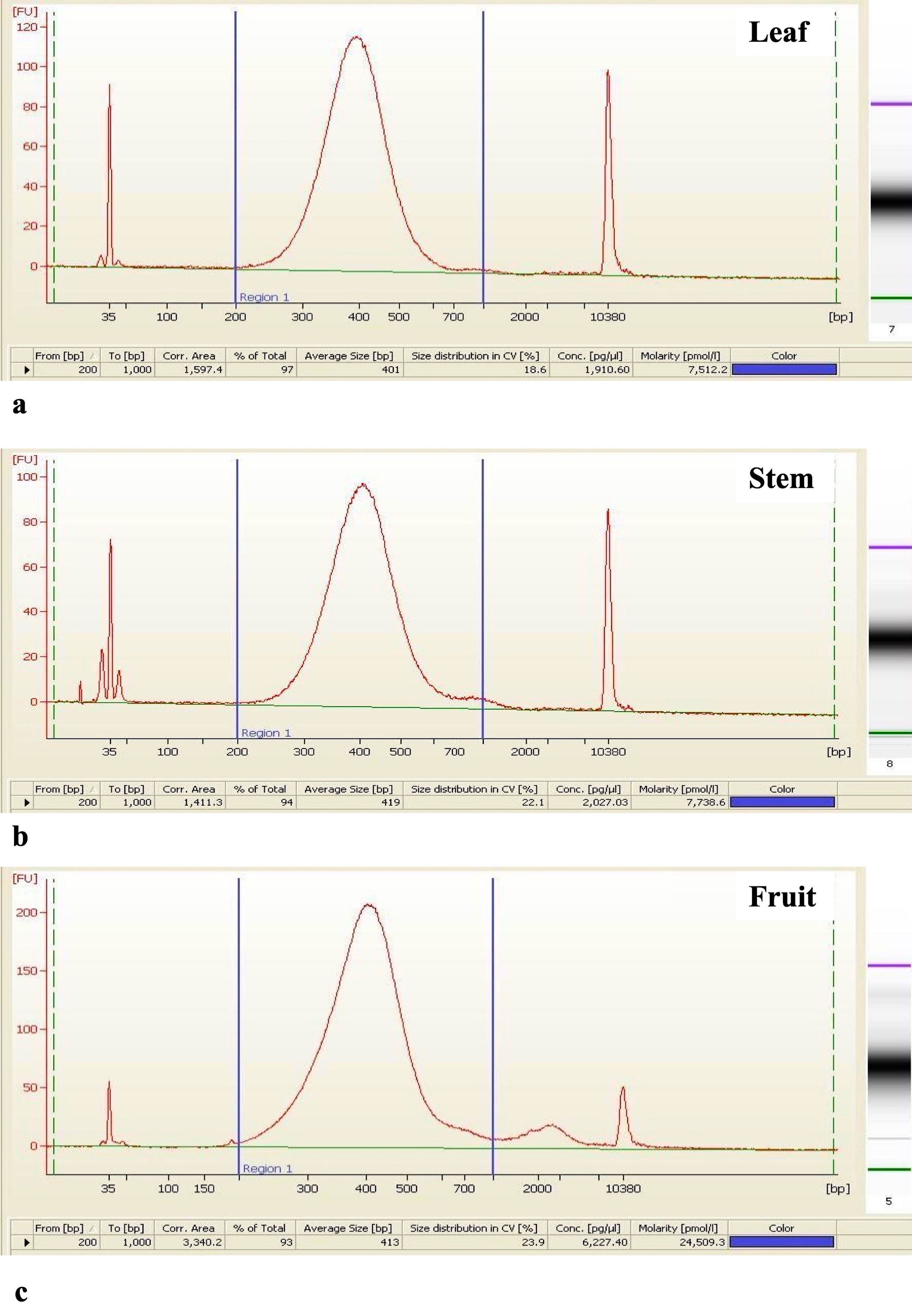


Supplementary Fig. 2. Library profile of leaf, stem and fruit on Agilent DNA HS Chip.


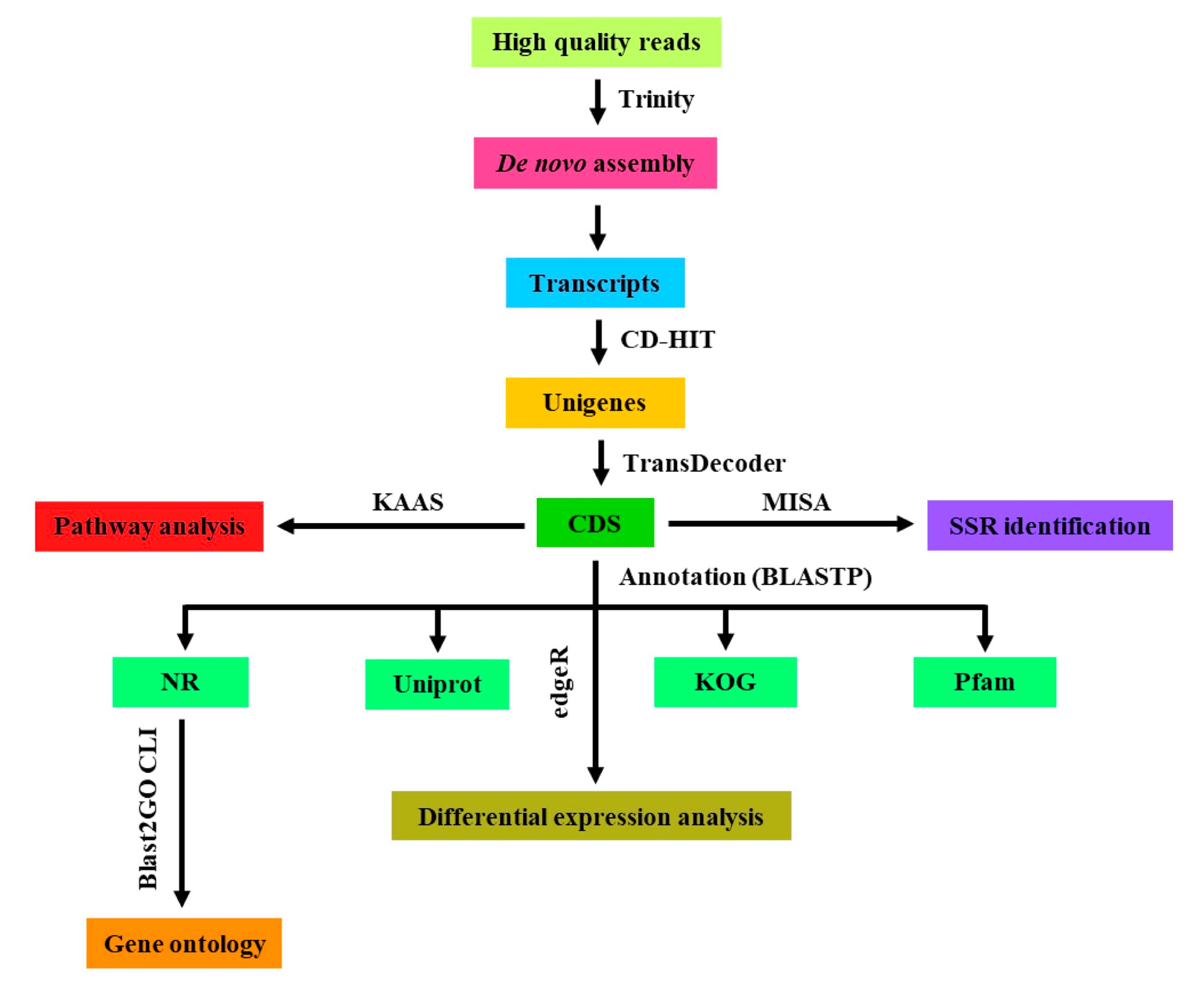


Supplementary Fig. 3. Bioinformatics Analysis Workflow.


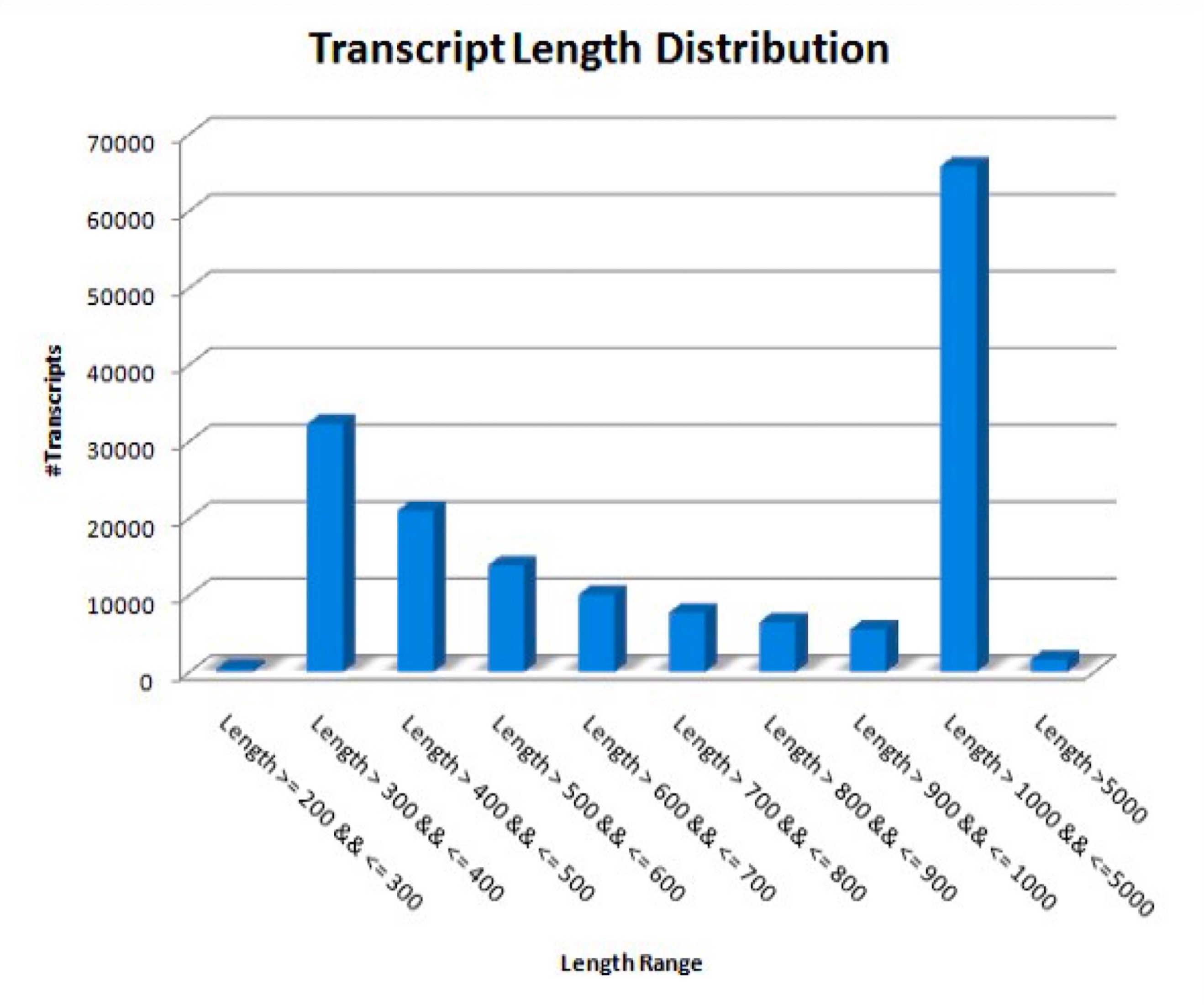


Supplementary Fig. 4. Distribution of transcripts was performed according to its length, where range of transcript is plotted in X-axis and number of transcripts is plotted in Y-axis.


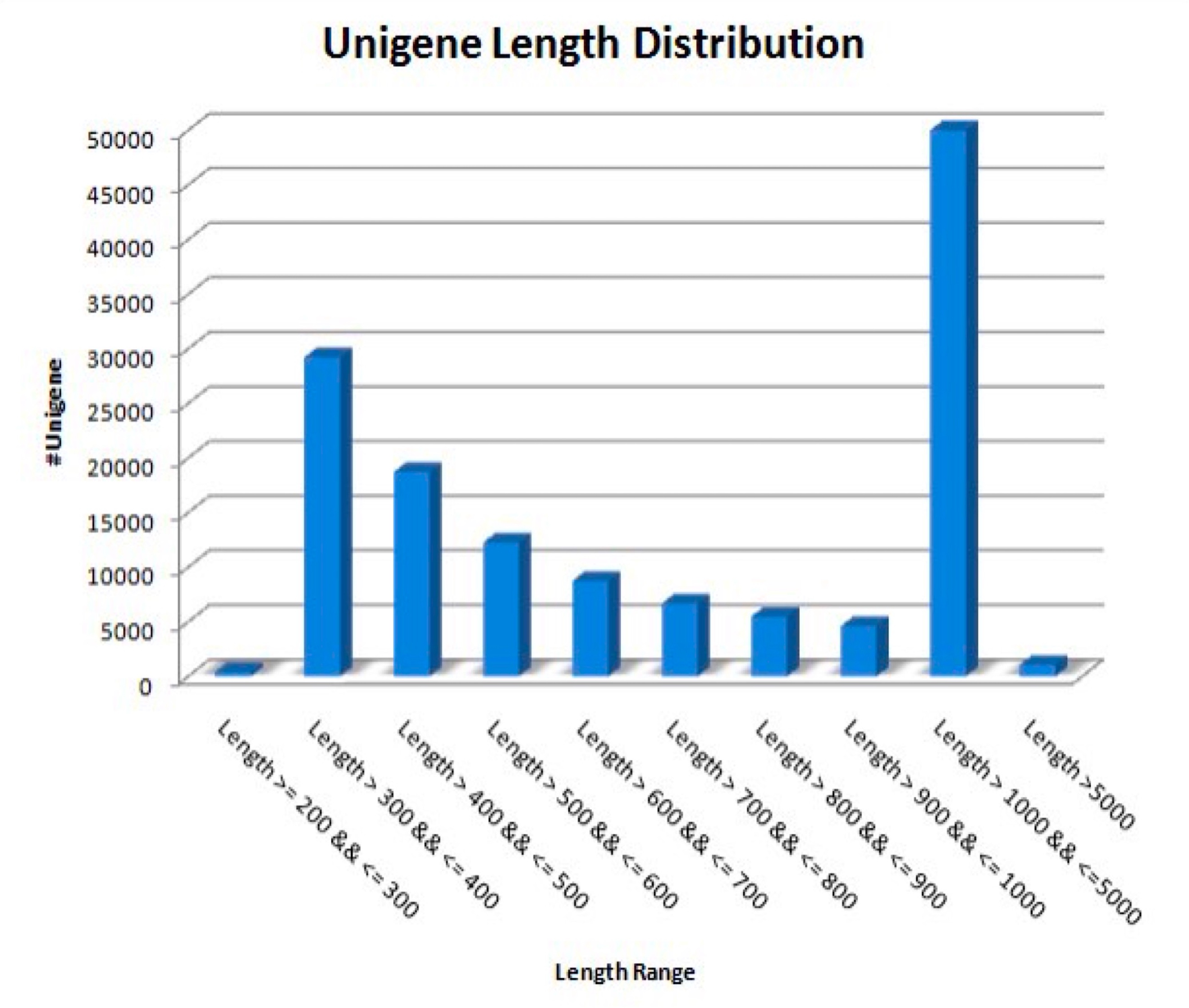


Supplementary Fig. 5. Distribution of unigenes was performed according to its length, where range of unigenes is plotted in X-axis and number of unigenes is plotted in Y-axis.


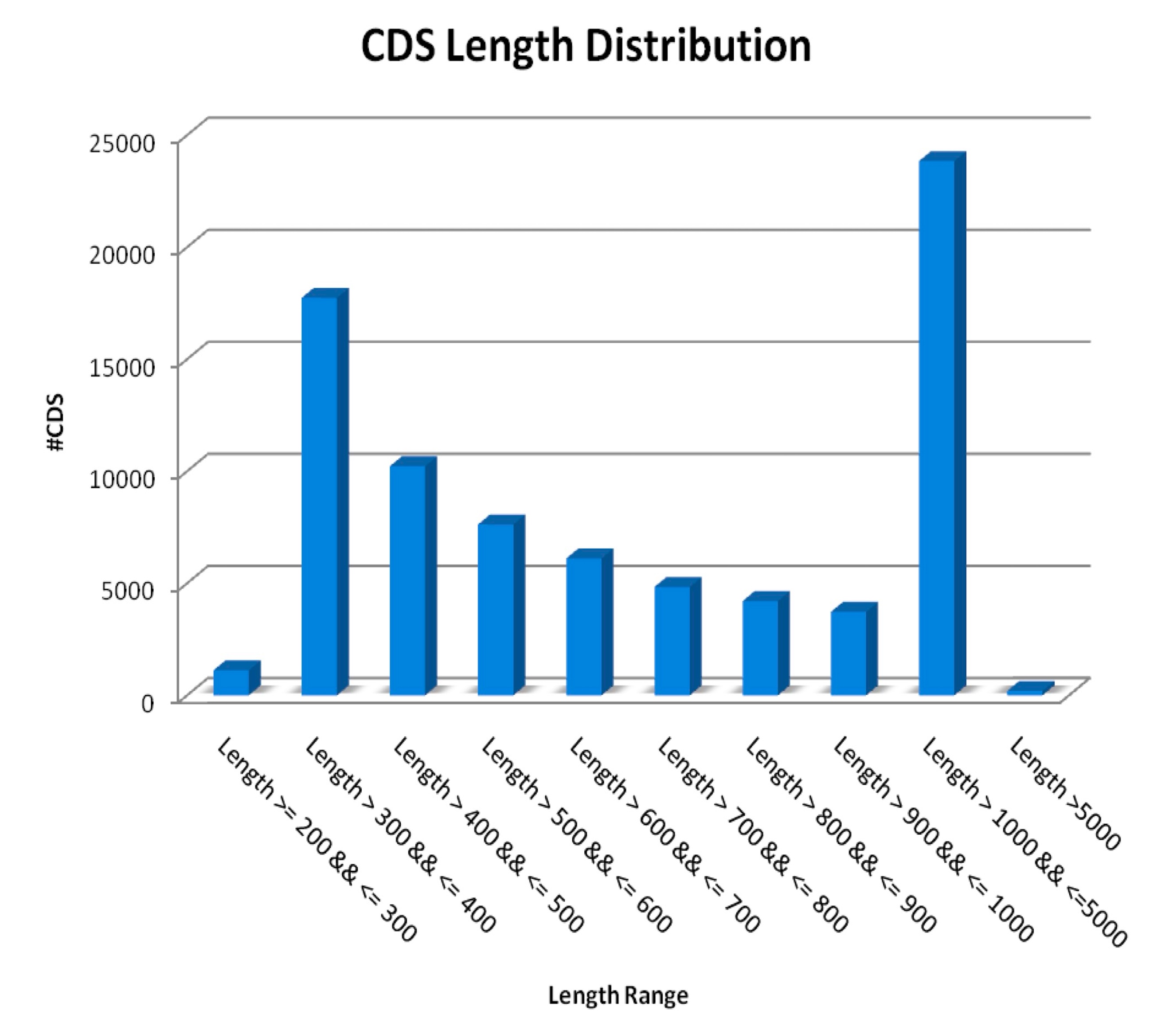


Supplementary Fig. 6. Distribution of CDS was performed according to its length, where range of CDS is plotted in X-axis and number of CDS is plotted in Y-axis.


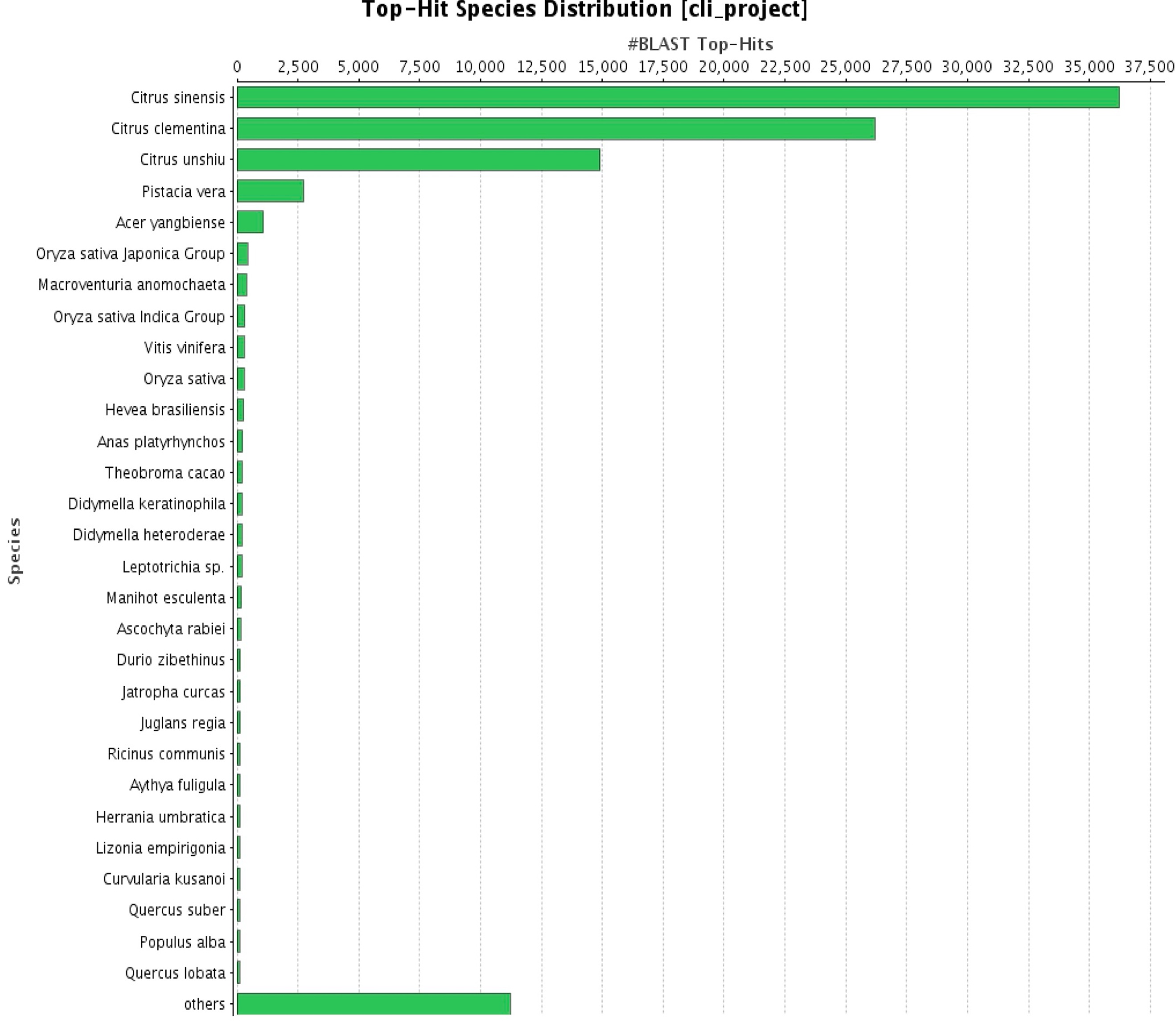


Supplementary Fig. 7. Functional Annotation of Predicted CDS. Ortholog assignment and mapping of the CDS to the biological pathways were performed using KEGG automatic annotation server (KAAS).


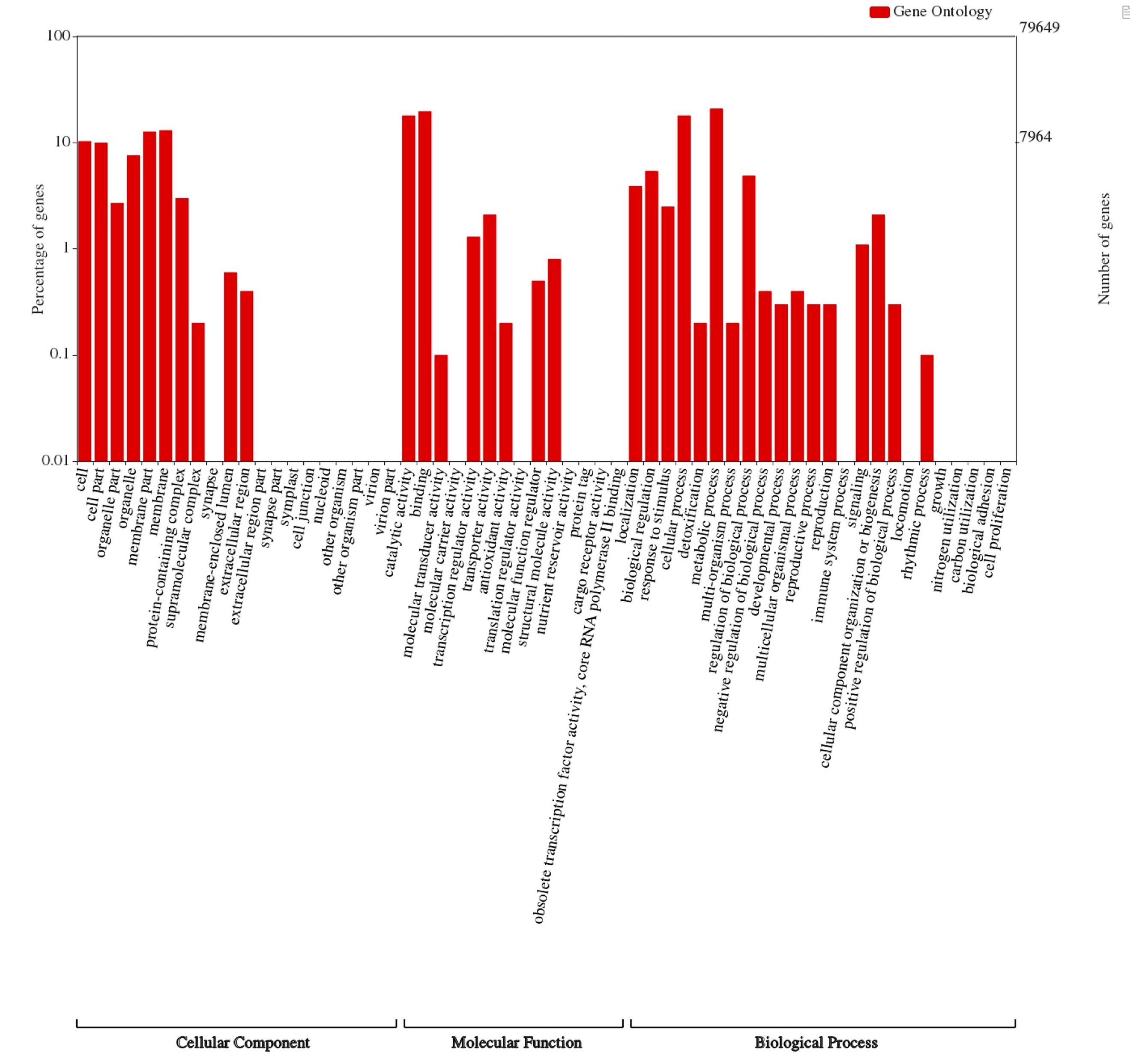


Supplementary Fig. 8. GO sequence distribution using WEGO is shown graphically.


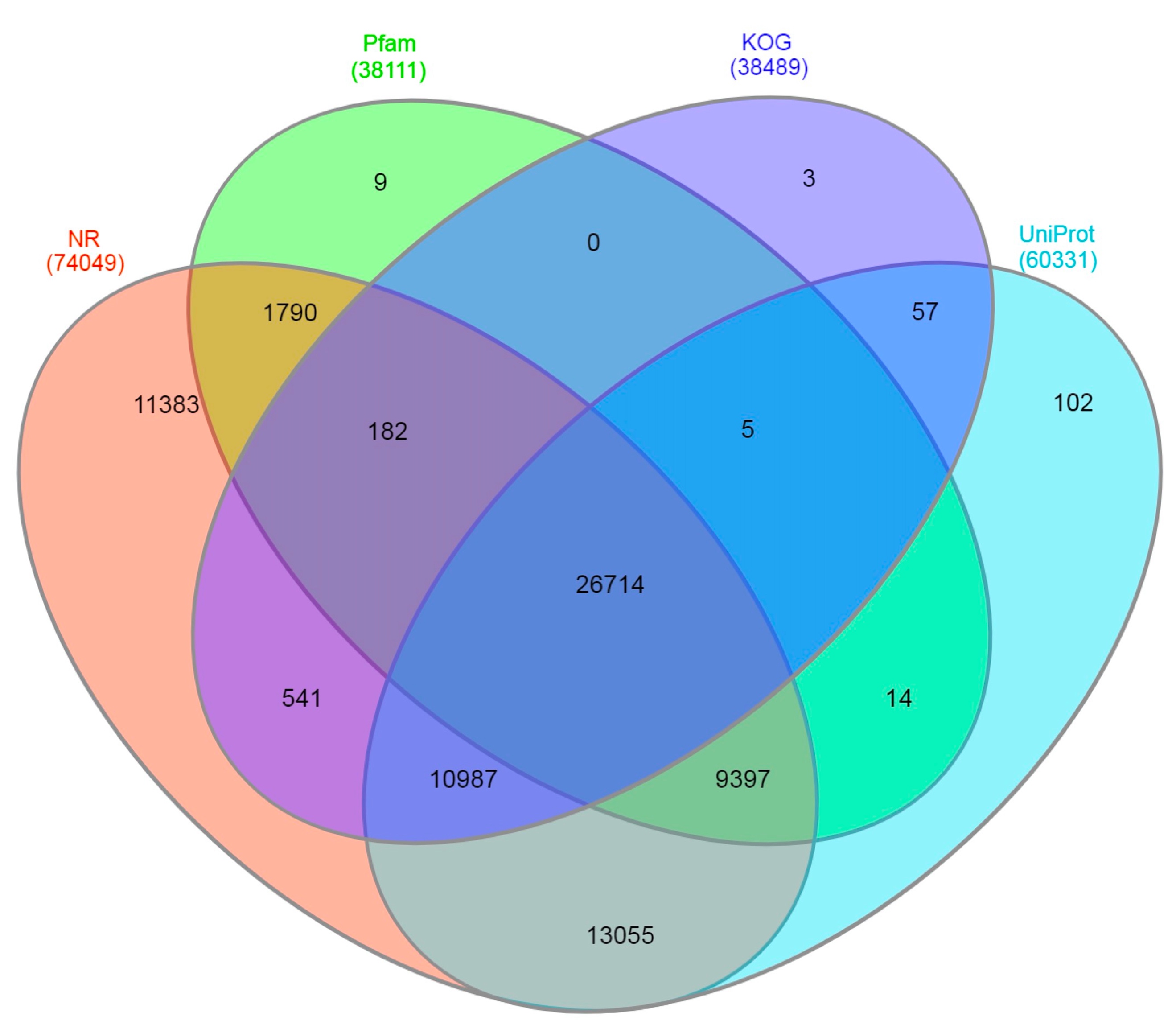


Supplementary Fig. 9. Venn diagram using InteractiVenn web server.


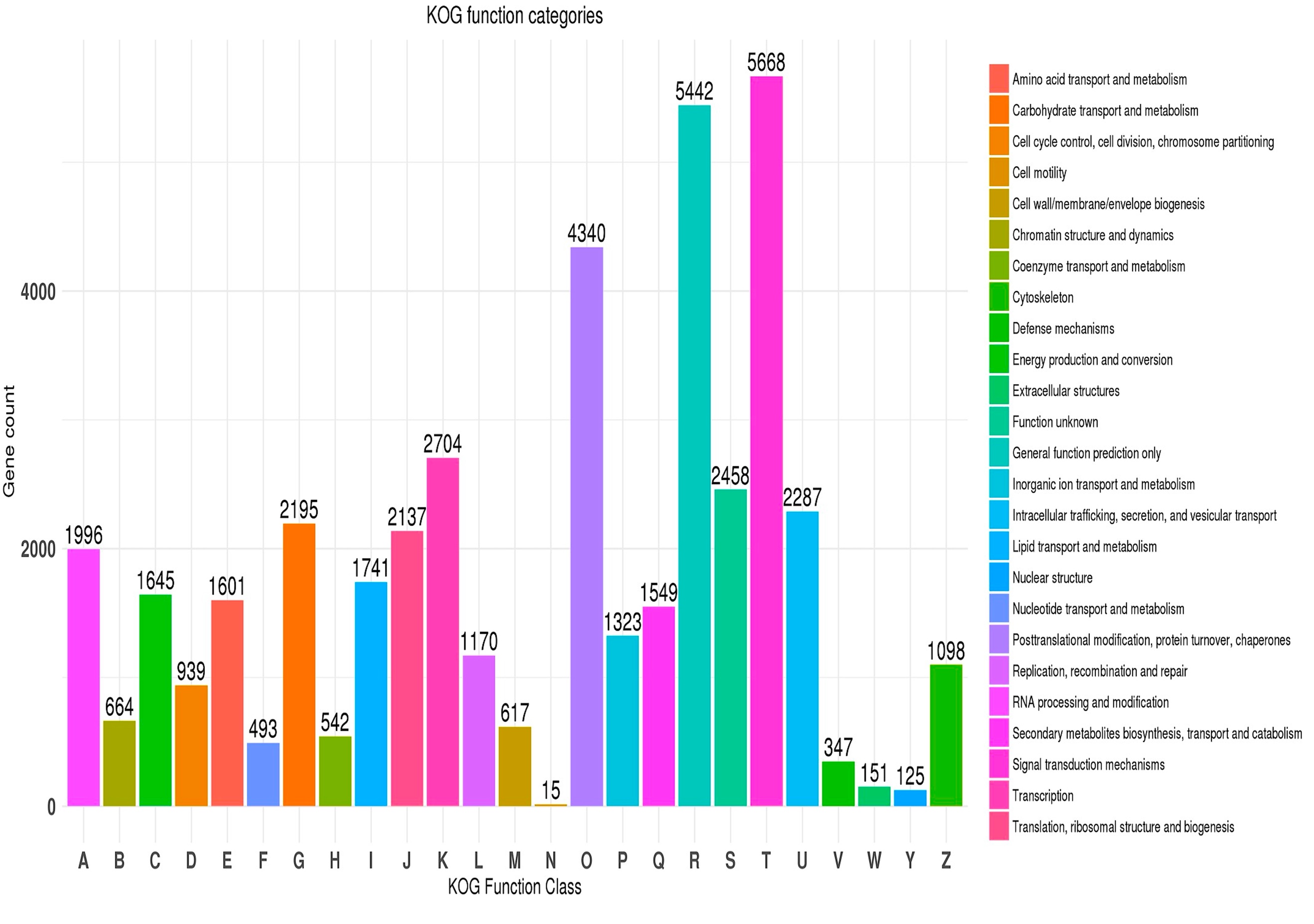


Supplementary Fig. 10. KOG classification for CDS. CDS were assigned at least one GO term (single CDS can have more than one GO terms).


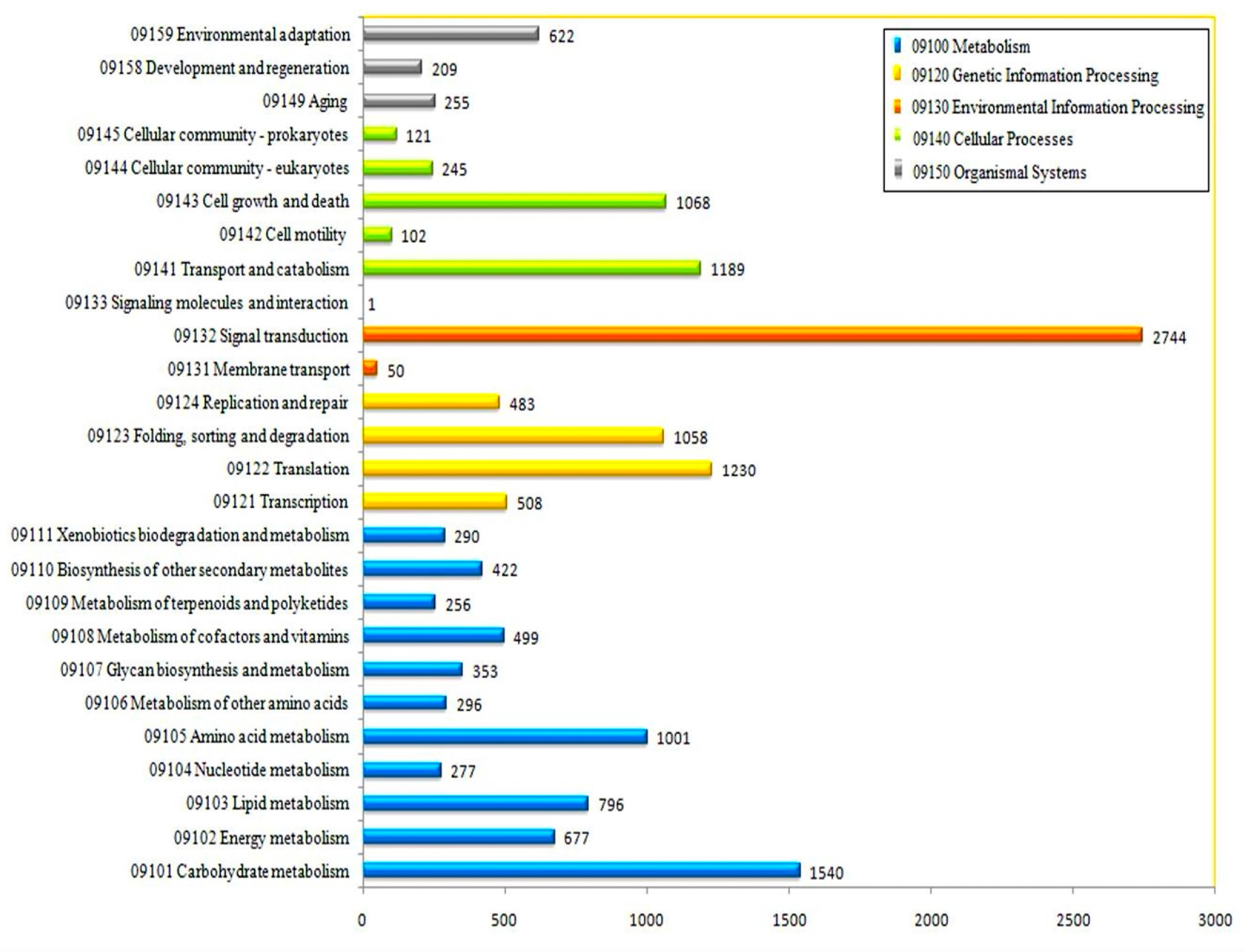


Supplementary Fig. 11. Distribution of CDS for KEGG pathway analysis.

Supplementary Fig. 12. Linear regression plot between WTS and RT-qPCR expression data based on the log2FC of six DEG identified in L vs S, L vs F and S vs F. Log2FC of WTS and RT-qPCR for the selected DEGs.

Supplementary Fig. 13. Comparative account of gene expression of berberine production pathway genes from the three samples of *Zanthoxylum armatum* at different seasons of the year.

Supplementary Fig. 14. Comparative account of gene expression of sanguinarine production pathway genes from the three samples of *Zanthoxylum armatum* at different seasons of the year.


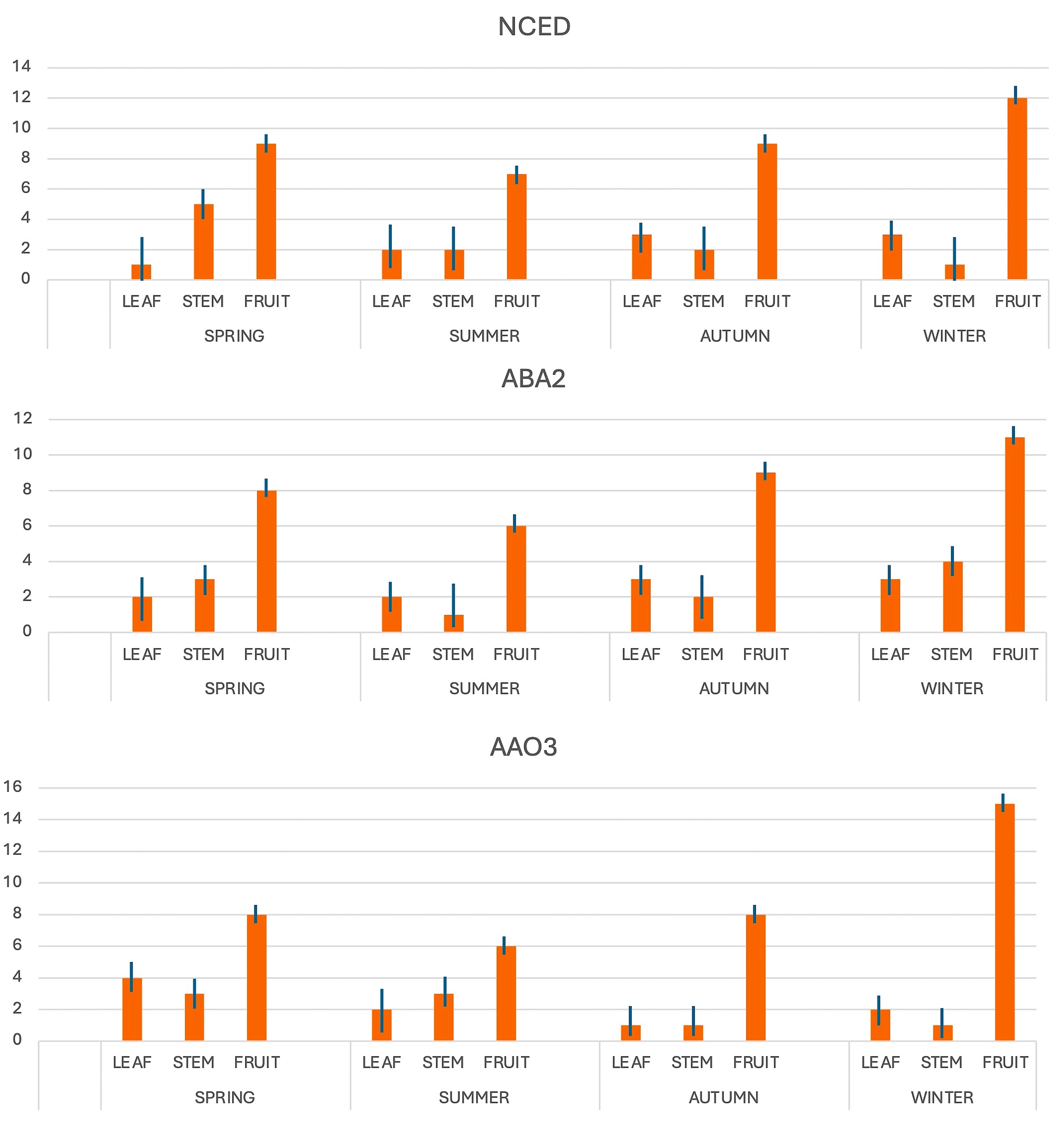


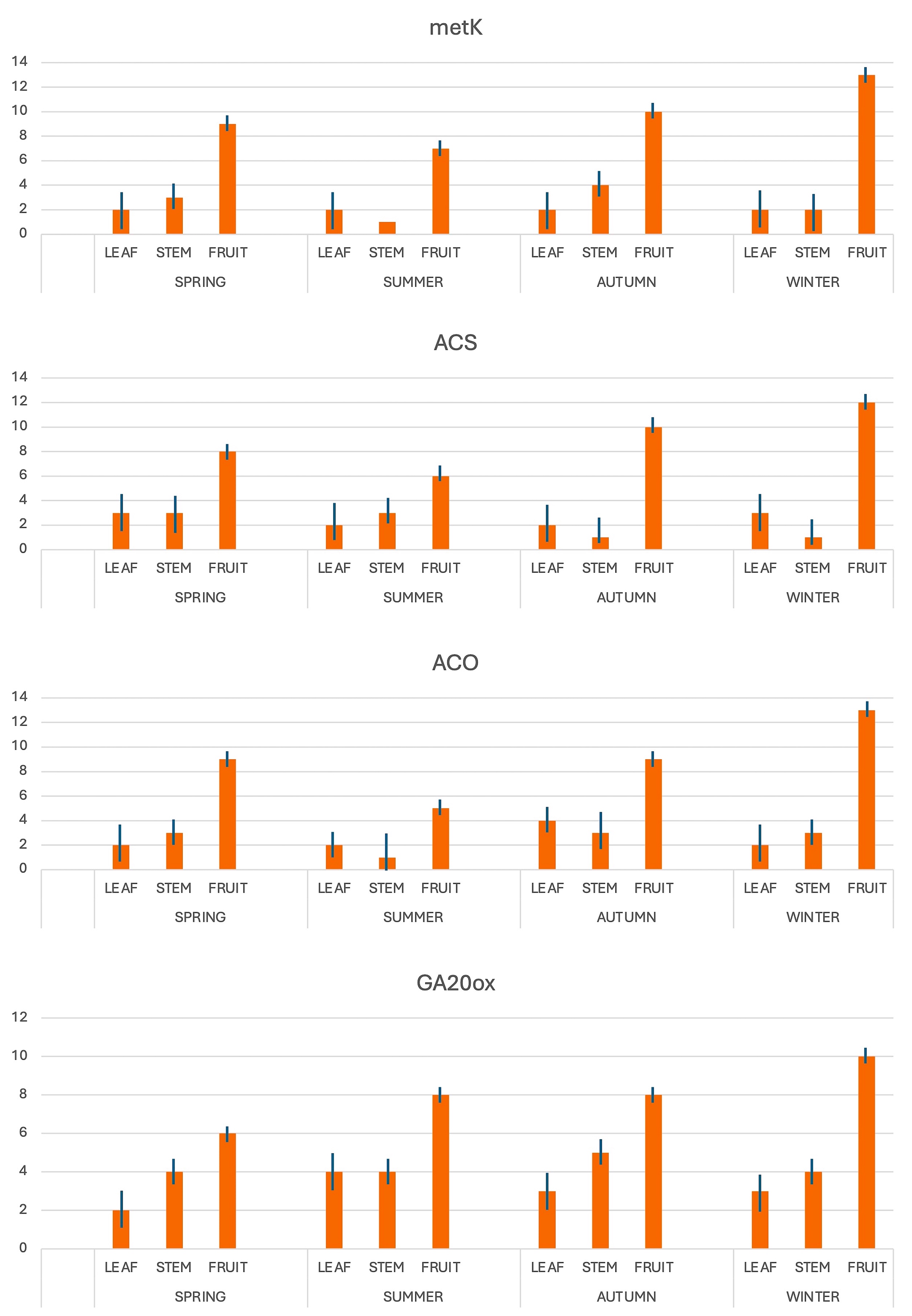


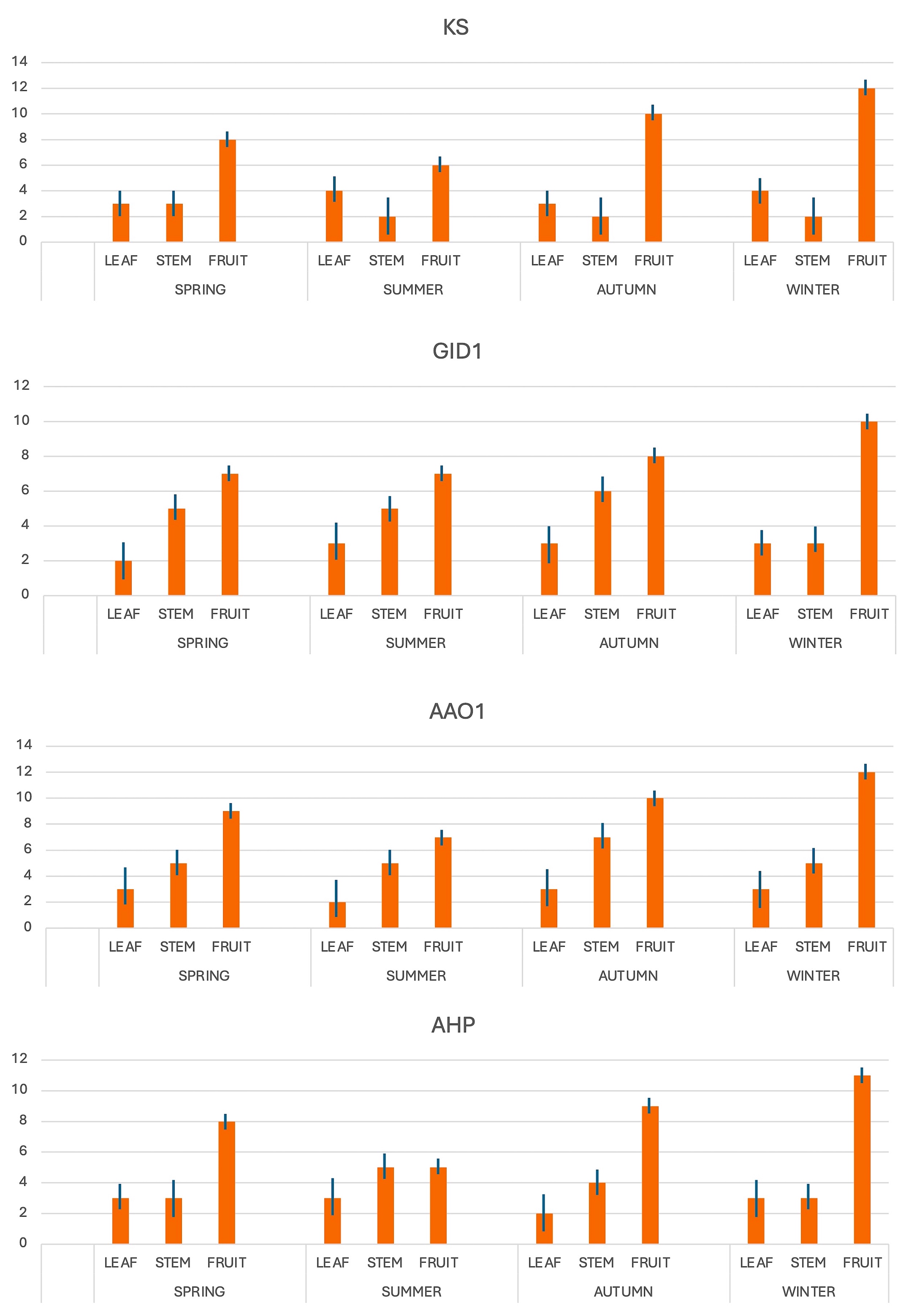


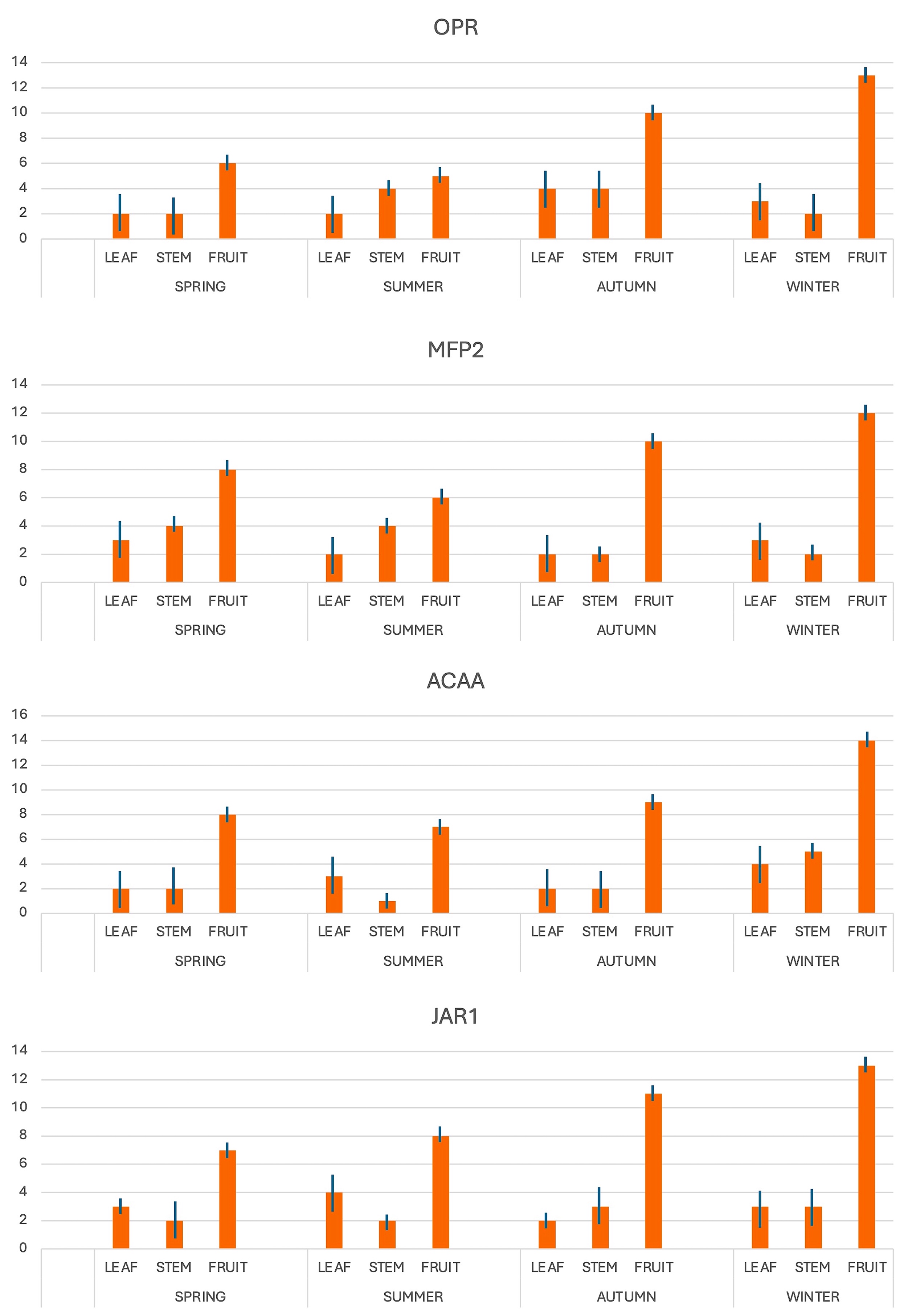


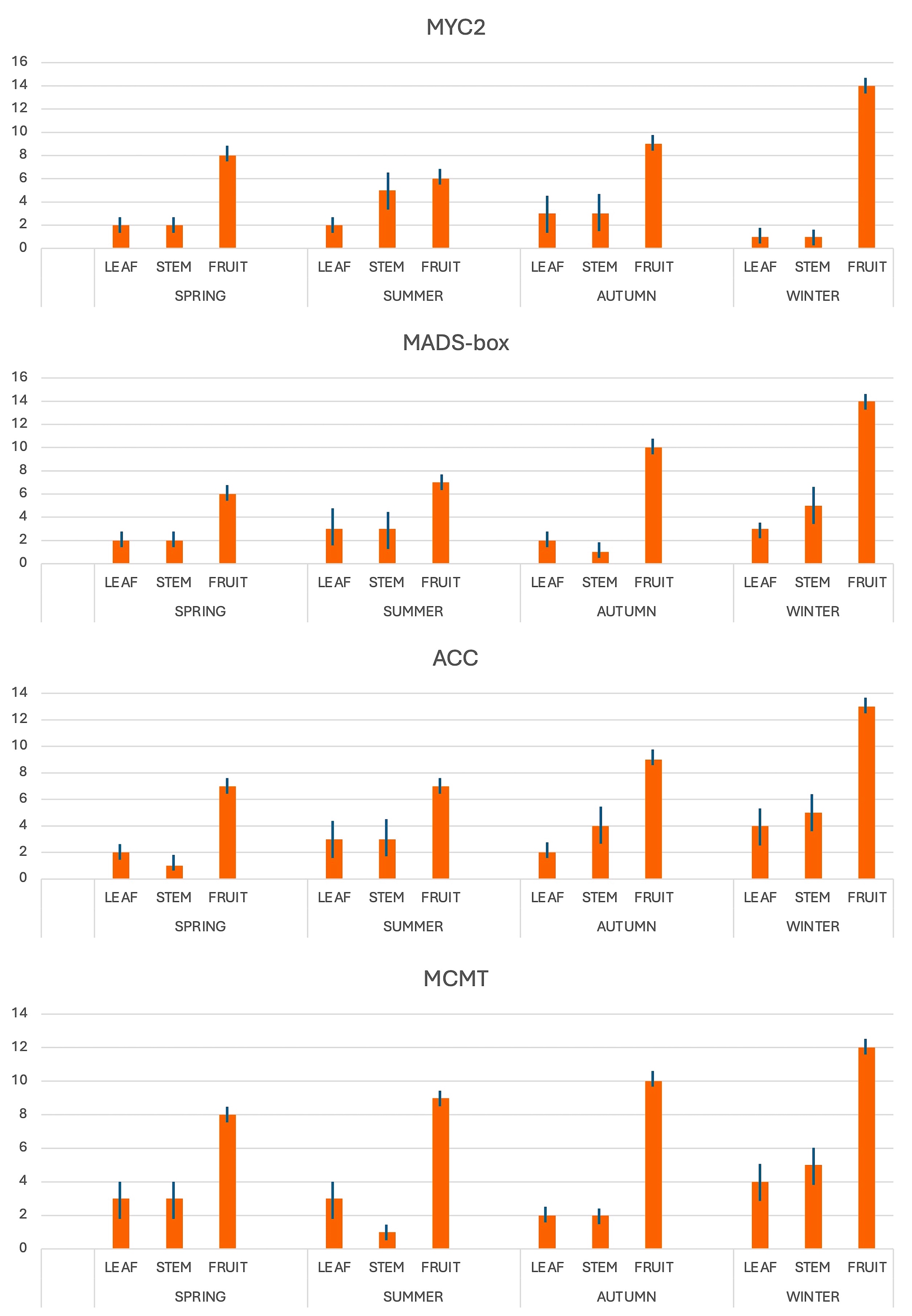


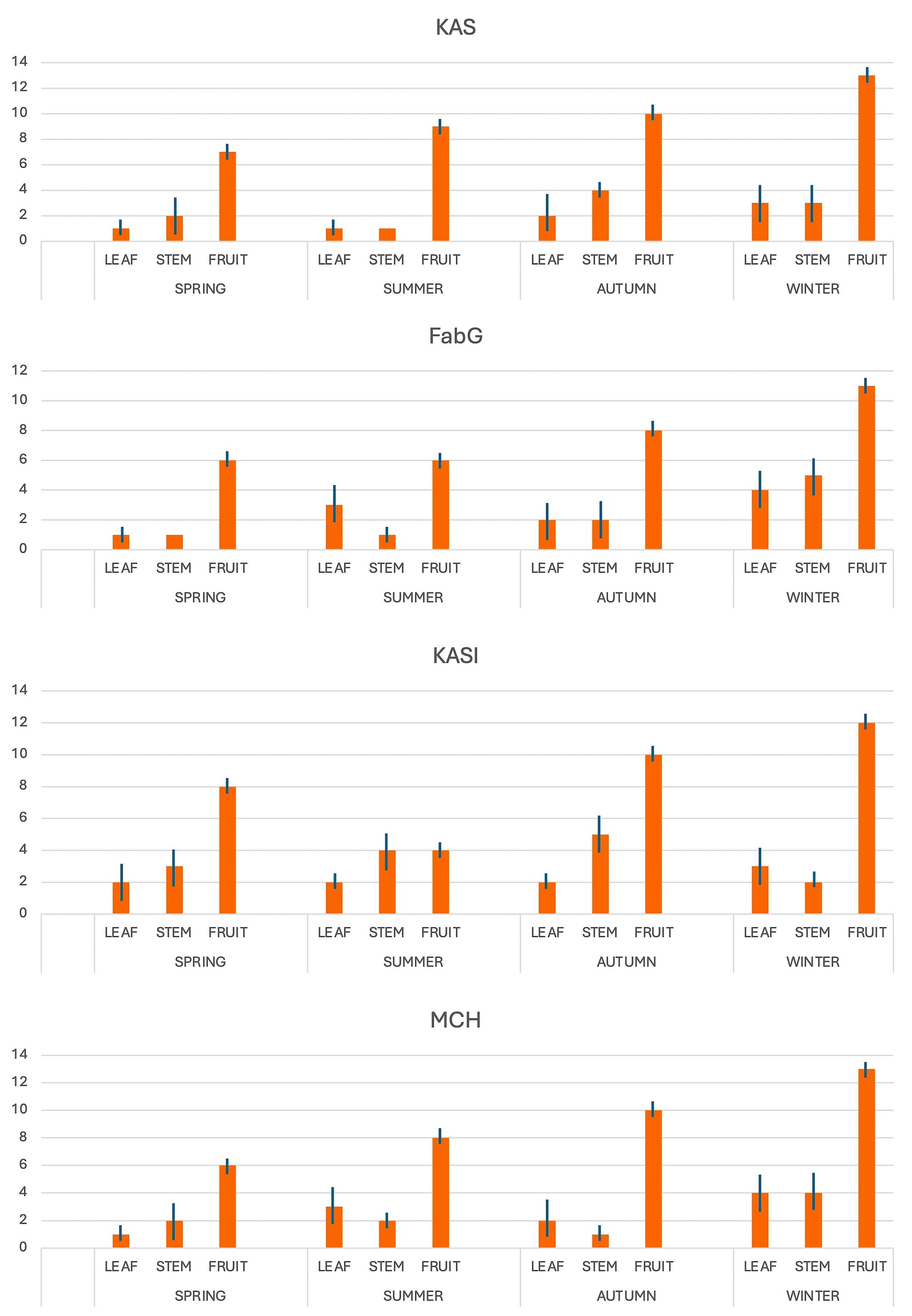


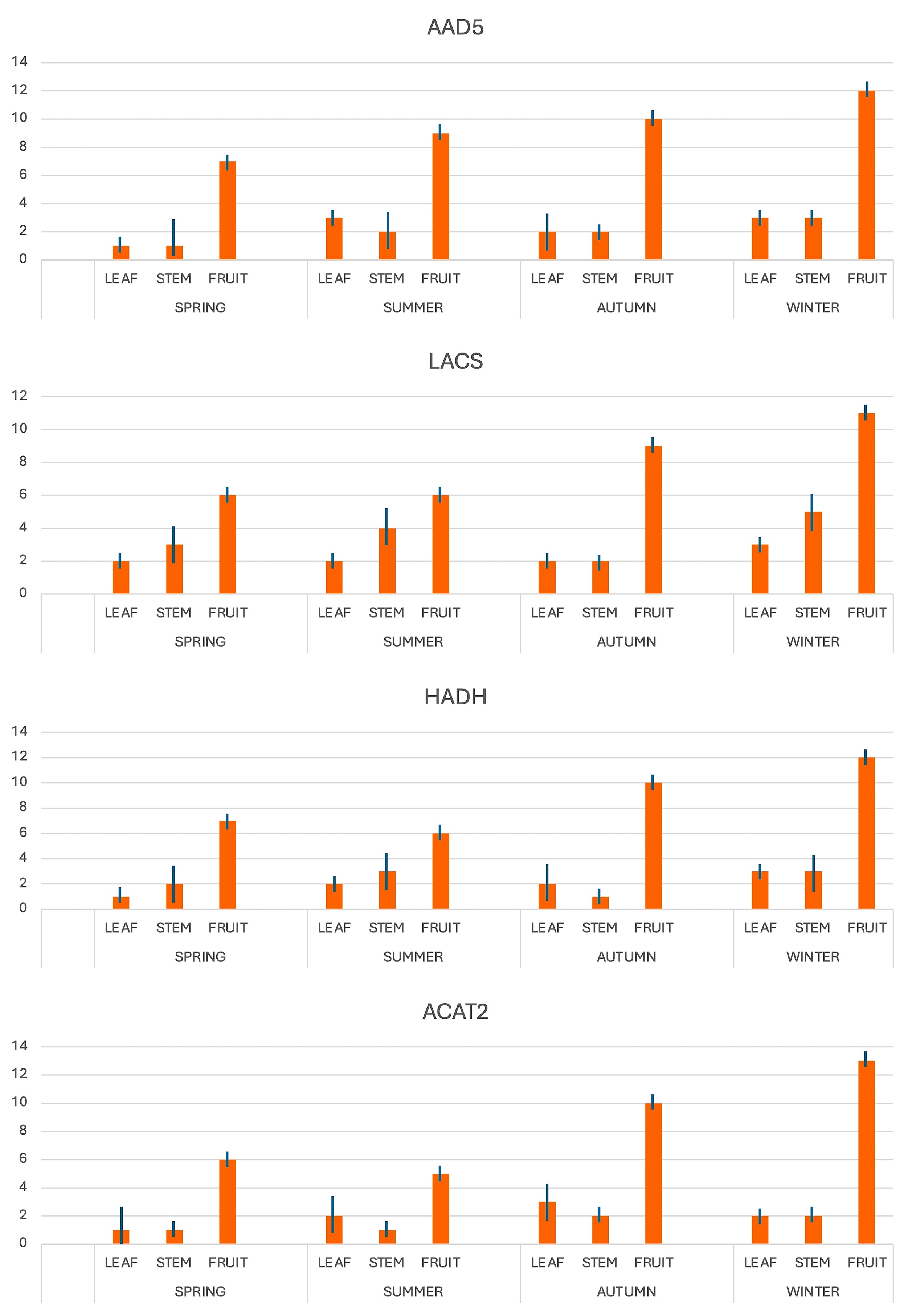


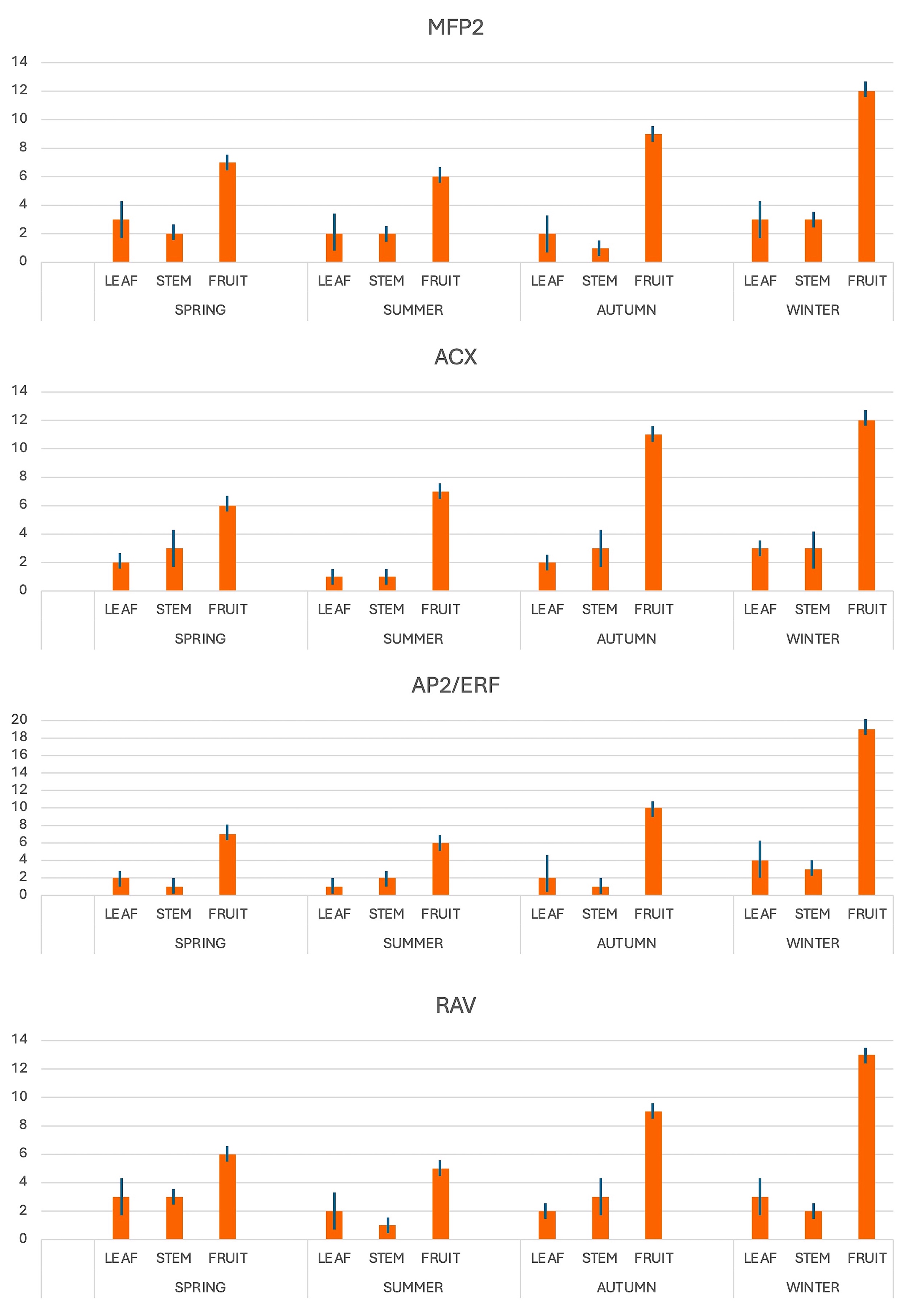


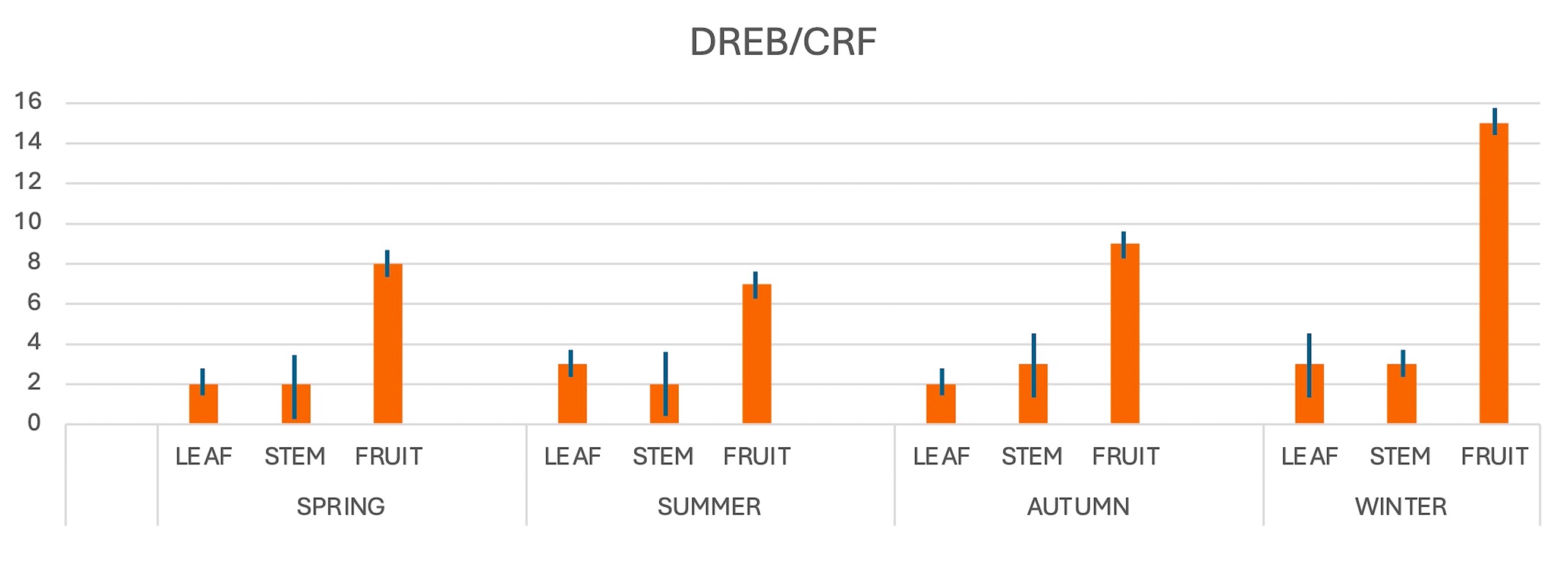


Supplementary Fig. 15. Relative expression of putative genes leading to the synthesis of various phytohormones and fatty acids which were identified from the assembled transcript library, along with the transcription factors (TFs) which are related to synthesis of phytohormones and fatty acids in *Zanthoxylum armatum* samples. Three sample types and four seasons were considered for the analysis. [A: ; B: ;


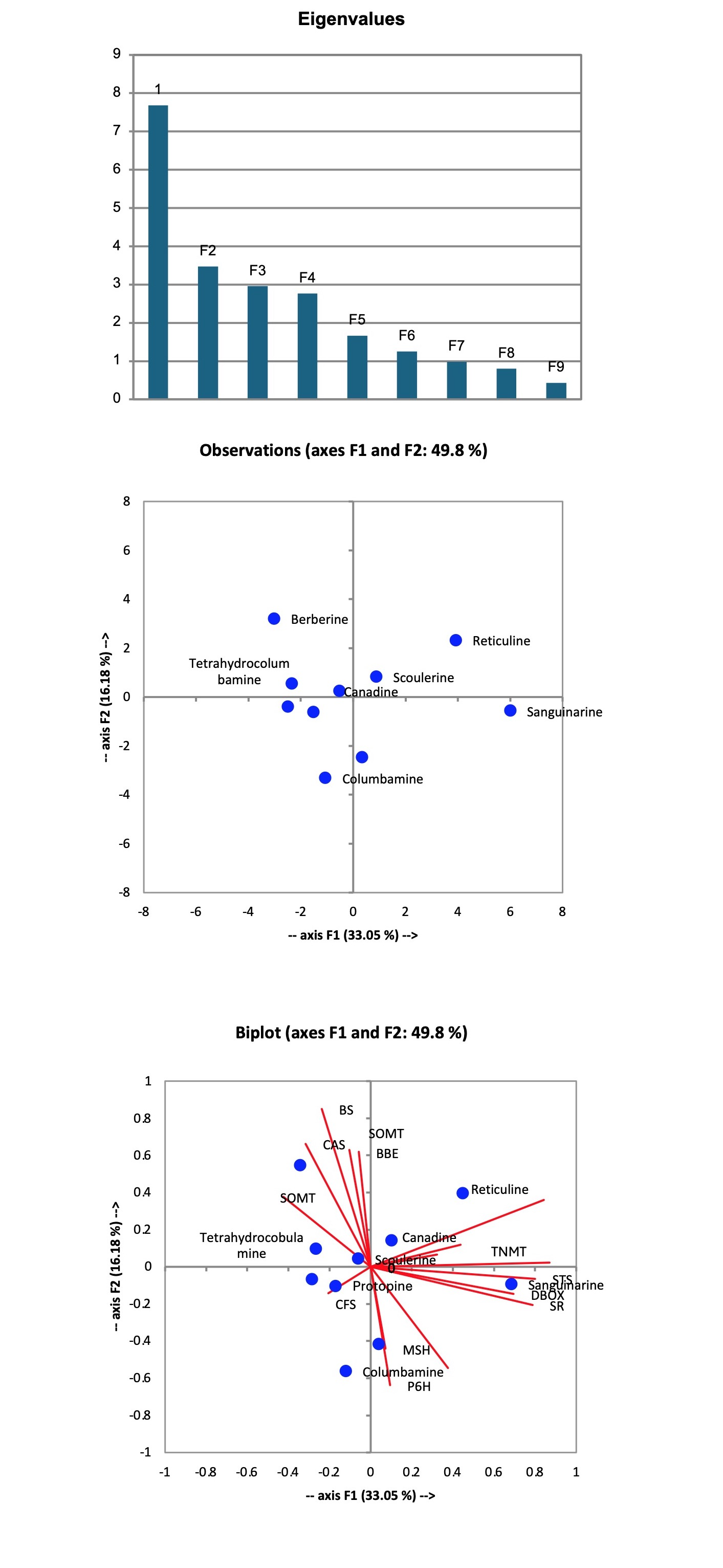


Supplementary Fig. 16. Principal Component Analysis (PCA for axes F1 and F2) for berberine and sanguinarine target compounds and the genes/transcripts involved in the pathway for production in *Zanthoxylum armatum*. [Data analysis was done using XLSTAT 7.5.2, uniform weighting and variance 1/n Pearson Correlation Coefficient].
